# Sex Differences in Piaget’s Water Level Tasks: A Systematic Review and Multiverse Meta-Analysis

**DOI:** 10.3390/jintelligence14100243

**Published:** 2026-10-03

**Authors:** Florence Dejardin, Sandra Oberleiter, Martin Voracek, Jakob Pietschnig

**Affiliations:** 1 Department of Developmental and Educational Psychology, Faculty of Psychology, University of Vienna, A-1010 Vienna, Austria; sandra.oberleiter@univie.ac.at; 2 Department of Cognition, Emotion, and Methods in Psychology, Faculty of Psychology, University of Vienna, A-1010 Vienna, Austria; martin.voracek@univie.ac.at

**Keywords:** water level task, Jean Piaget, spatial abilities, spatial perception, sex differences, multiverse meta-analysis, specification-curve analysis, combinatorial meta-analysis

## Abstract

Jean Piaget developed the water level tasks (WLTs) to investigate emerging Euclidean spatial concepts of horizontality invariance in children, expecting universal mastery by about age nine. However, it has been shown that even adults have difficulties solving these tasks, with men on average outperforming women. While this effect has repeatedly been replicated, its generalizability remains unclear. Here, we meta-analytically synthesized the retrievable evidence for sex differences in WLT performance (1964–2025, 267 samples, *N* = 38,000+). Potential effect moderators were examined via subgroup analysis, precision-weighted meta-regression, and (multiverse-style) specification-curve and combinatorial meta-analysis, accounting for numerous reasonable decisions regarding which data to meta-analyze (data multiverse) and how to synthesize them (model multiverse). Results showed medium-to-large-sized male advantages in WLT performance (*d* = 0.50; 95% CI [0.47, 0.54]) that increased with age. Effect sizes declined across publication years, particularly among adolescents, and were smaller in more recent birth cohorts. Multiverse-style meta-analyses revealed a remarkable generality of this effect across sample characteristics, test variants, and analytic approaches, with 94% of 13,204 models yielding nominally significant male advantages. These findings indicate moderate-to-large, robust, yet cross-temporally declining sex differences in WLT performance, challenging Piaget’s idea of a universal understanding of horizontality invariance by late childhood.

## 1. Introduction

In *The Child’s Conception of Space*, the Swiss developmental psychologists Piaget and Inhelder (1948/1956) examined the emergence of spatial cognition within the framework of Piaget’s stage theory of cognitive development. According to these ideas, every typically developing child progresses through a sensorimotor, a pre-operational, a concrete operational, and a formal operational stage in a hierarchical manner as their general system of thought evolves (Piaget, 1962). In this work, the development of topological, projective, and Euclidean spatial concepts was investigated with a series of experiments. To assess Euclidean concepts, horizontality invariance was deemed a central principle, namely, the understanding that the surface of liquids invariantly remains horizontal, enabled by the ability to rely on an external frame of reference.

To examine its development, the so-called water level tasks (WLTs) were developed, which require drawing the water surface into empty tilted containers. Based on children’s and adolescents’ WLT responses, the progressive development of a stable coordinate system required for representing orientation and movement of objects in space during the late concrete operational stage was proposed, culminating in the immediate prediction of the invariant horizontal at about age nine (for a detailed description of the stages in the development of WLT performance postulated by Piaget and Inhelder (1948/1956), see Liben (1991b). Subsequently, the horizontality principle was assumed to be directly applied to all situations from this age onward due to a complete organization of concrete operational thought (Piaget & Inhelder, 1948/1956), thus implying that every typically developing individual older than about nine years of age should be able to solve WLTs correctly. However, subsequent research has challenged this idea.

### 1.1. Sex Differences in Adult WLT Performance

Subsequent accounts have shown that even adults experience considerable difficulty solving these seemingly simple tasks (Rebelsky, 1964). Moreover, evidence also suggests performance advantages for men compared to women. Over the past decades, these sex differences have repeatedly been replicated across different adaptations of the original task design. Across studies, WLT implementations varied considerably in terms of (i) item numbers, ranging from one (e.g., Cashdan et al., 2012) to more than 200 items (e.g., Rilea, 2008); (ii) vessel shapes, such as rectangular stimuli (e.g., S. A. Sorby & Veurink, 2019), Florence flasks (e.g., Tran & Formann, 2008), or bottles (e.g., Sholl & Liben, 1995); and (iii) response modalities, including drawing water lines (e.g., Meneghetti et al., 2021), multiple-choice formats (e.g., Nordvik & Amponsah, 1998), or adjusting physical models (e.g., E. Geiringer, 1976). However, despite this heterogeneity, male advantages have been observed in children (e.g., Merrill et al., 2016), adolescents (e.g., Rilea, 2008), and adults alike (e.g., Tomai et al., 2024).

### 1.2. The WLT and Spatial Ability

Since their development, different WLT versions have become increasingly popular as measures of spatial perception (e.g., Merrill et al., 2016; Nordvik & Amponsah, 1998), i.e., the ability to determine spatial relationships with respect to the orientation of one’s own body despite distracting information (Linn & Petersen, 1985). However, WLTs have also been used to measure extrinsic-static skills, which require an understanding of the position of objects relative to a reference frame and to other objects (Newcombe & Shipley, 2014), or to assess frame of reference illusions (Buckley et al., 2018). Although terminologies vary across studies and theoretical accounts, WLT scores have consistently been linked to the perceptual component of spatial cognition (Uttal et al., 2013a).

### 1.3. Sex Differences in Spatial Cognition

From an educational-developmental perspective, studying cognitive sex differences can provide valuable insight into sources of performance disparities (manifested as distributional tail ratios; Voracek et al., 2013) and inform the development of targeted interventions (Halpern, 2013). Within this context, spatial skills warrant particular attention, because they play a key role in the development of proficiency in domains related to science, technology, engineering, and mathematics (STEM; Kell & Lubinski, 2013; Khine, 2017). Longitudinal investigations have identified performance in spatial measures as a predictor of later STEM attainment and achievement (Shea et al., 2001; Uttal et al., 2013b; Wai et al., 2009; Webb et al., 2007).

More recent meta-analyses further point to moderate-to-large associations between spatial and mathematical skills (Atit et al., 2022; Xie et al., 2020), with converging evidence from studies reporting WLT results (Scott et al., 2023; Voyer, 1998). Moreover, WLT performance has been associated with expertise in the geosciences (Liben & Titus, 2012; McNeal et al., 2024) and other STEM-related disciplines (e.g., Nordvik & Amponsah, 1998; Tomai et al., 2024). Against this background, sex differences in spatial abilities have been discussed as one potential driver of the persistent underrepresentation of women in STEM education and occupations (Baker & Cornelson, 2018; Signorella & Liben, 2024), with women making up fewer than one in four employees in high-technology sectors within the European Union (EU) in 2025 (Eurostat, 2026). Therefore, sex differences in WLT performance may represent one of many factors contributing to these disparities. Given the substantial heterogeneity in study designs and sample compositions, a comprehensive, up-to-date meta-analytic investigation is needed to clarify the strength and pervasiveness of this sex gap.

### 1.4. Prior Related Meta-Analyses

Previous meta-analyses have consistently documented moderately sized sex differences favoring men in spatial perception (i.e., extrinsic-static spatial ability) tasks in general (Linn & Petersen, 1985; Voyer et al., 1995) and WLTs in particular, with summary effect sizes of *d* = 0.42 (*k* = 62; Voyer et al., 1995) in a published meta-analysis, as well as *d* = 0.60 (*k* = 140, Pavlovic et al., 2008), and *d* = 0.55 (*k* = 157; Pietschnig & Voracek, 2011) in two unpublished meta-analyses (i.e., conference presentations). Moreover, previous syntheses have shown that these sex differences increase with age (Linn & Petersen, 1985; Pietschnig & Voracek, 2011; Voyer et al., 1995), while remaining relatively stable between cohorts (Pietschnig & Voracek, 2011). However, all of these prior related accounts are outdated and, naturally so, lack the level of methodological rigor and evidential value of contemporary techniques of quantitative evidence synthesis.

### 1.5. Present Meta-Analysis

Despite more than six decades of accumulated WLT research, little is known about the generality of the effect strength of WLT-based sex differences. Specifically, it remains unclear whether these differences vary as a function of moderators, such as the administered WLT version, sample characteristics, or analytic approaches. Moreover, the strength of observed effects across various empirical disciplines, including psychological science, has been shown to be susceptible to cross-temporal decline phenomena (Pietschnig et al., 2019; Protzko & Schooler, 2017). In light of such decline effects, as well as a potential shift toward more egalitarian socialization practices (D. I. Miller et al., 2018), an up-to-date examination of potential changes in the strength of these sex differences over time is expedient.

Here, we meta-analytically synthesize all retrievable reports on sex differences in WLT performance. To assess the generalizability of these effects, we examine potential moderator influences in dedicated subgroup analyses and meta-regressions. Furthermore, we present multiverse-style analyses to account for the influences of all (reasonable) ways of which data to include and how to meta-analyze them. Finally, we investigate potential confounding influences of publication bias-related mechanisms by using several detection methods for dissemination bias. In all, this meta-analysis provides the so far most comprehensive and robust synthesis of the extant evidence on sex differences in WLT performance across more than six decades of research.

## 2. Materials and Methods

This meta-analysis, including its analysis plan, was preregistered on 27 March 2024 at the Open Science Framework (https://osf.io/zdwfh (accessed on 28 August 2026)), before accessing any data. All preregistration deviations are documented in the online Supplementary Materials (available at https://osf.io/6fwsa/files/ndfpt (accessed on 28 August 2026); see Table S1.1). The PRISMA 2020 checklist (Page et al., 2021) is provided in the online Supplementary Table S1.

### 2.1. Literature Search

First, all studies included in the two most recent (but unpublished) meta-analyses on sex differences in WLT performance (Pavlovic et al., 2008; Pietschnig & Voracek, 2011) were obtained. Second, to identify additional relevant studies, we conducted extensive literature searches in databases with coverage of published (as well as unpublished) studies (Google Scholar, ISI Web of Science, Open Access Theses and Dissertations, ProQuest, PsycInfo, PubMed, Scopus). The following search string was used: “water level task” OR “water level test” OR “water level experiment” OR “water level representation” OR “water line task” OR “water level concept” OR “liquid horizontality” OR “Piaget water” OR “Piaget horizontal*” OR “Piaget spatial*” OR “Wasserspiegelaufgabe”. Third, we conducted cited-reference searches of the published meta-analyses on this topic (namely, Linn & Petersen, 1985; Signorella & Jamison, 1986; Voyer et al., 1995). Finally, we screened the reference lists of all studies deemed eligible for inclusion for further potentially includable studies.

Titles and abstracts of the resulting records were screened for eligibility and full texts of potentially relevant studies were subsequently obtained. Studies with abstracts or full texts in languages other than English, German, or French (18 records in total) were translated using DeepL (https://www.deepl.com/en/translator; accessed on 28 August 2026). Of note, although this approach allowed studies to be considered irrespective of publication language, the use of automated translation cannot entirely rule out potential language bias (Balk et al., 2013). In total, our search yielded 2387 records. After removing duplicates, 1565 titles and abstracts were screened, and the full texts of 1057 studies were assessed for eligibility (see Figure 1). Details of the excluded studies and the respective reasons for exclusion are provided in the online Supplementary Materials at https://osf.io/6fwsa/files/udpvn (accessed on 28 August 2026). These literature searches were conducted from April to June 2024 and updated and finalized in May 2025.

### 2.2. Inclusion Criteria

To be eligible for inclusion in the present meta-analysis, studies had to meet nine inclusion criteria: (i) A type of WLT was used, and (ii) sex differences were reported or sufficient statistical information was provided to compute effect sizes. Furthermore, (iii) samples consisted of healthy participants, (iv) samples were independent from each other (i.e., did not include overlapping participants), and (v) the average cognitive ability of the investigated sample did not exceed two standard deviations above or below the population mean to avoid ceiling or floor effects. Regarding the WLT version used, (vi) the test presented either a two-dimensional drawing (paper-and-pencil or computer-based), a photograph, or real vessels as stimuli. Test responses (i.e., the indication of the water level) were (vii) produced by hand-drawing the water line (pen-and-paper version), selecting from response alternatives (multiple-choice version), adjusting a model of the water level (e.g., using a ruler or a thread), or adjusting a model of the vessel. In cases where a reference line was provided, (viii) it was horizontal. Finally, (ix) no training or interventions were administered before testing.

### 2.3. Coding

For the 157 independent samples included in the two most recent meta-analyses (coded by SP; Pavlovic et al., 2008, and JP; Pietschnig & Voracek, 2011), coding was independently repeated by one researcher (FD). Agreement percentages between coding runs ranged from 92 to 100 percent across variables. Title and abstract screening and coding of additional studies were conducted by FD, and any ambiguities were discussed and resolved in consultation with JP. All coded information is provided in the online Supplementary Materials (see https://osf.io/6fwsa/files/ndfpt (accessed on 28 August 2026)).

In case of missing information, requests were sent to the corresponding authors of the primary studies via email. If no response was received, reminders were sent after two and four weeks. If the information required to calculate effect sizes (i.e., the respective sex difference) was not provided in a report and the corresponding authors did not respond two weeks after the final reminder, studies were excluded from further analyses. From a total of 133 records with missing information required to calculate an effect size, authors of 68 primary studies were contacted. Of the 36 authors who responded, 13 provided the requested information.

### 2.4. Data Analysis

Across studies, the classification of participants as male or female followed the reporting used by the original study authors (none of the included studies reported data on nonbinary or otherwise unclassified participants). If not reported in the primary studies, standardized mean sex differences were calculated as Cohen’s *d*, with positive values indicating advantages of men. The resulting effect sizes were weighted according to study precision (i.e., inverse effect size variances) and synthesized via restricted maximum likelihood (REML) random-effects meta-analyses (Viechtbauer, 2010).

To facilitate interpretability of the results, the obtained summary effect was transformed to Cohen’s *U*_1_, representing the proportion of nonoverlap between men and women relative to the total area under these two distributions (Gyimesi et al., 2025). Additionally, to account for group differences in the distributional tail regions, the summary effect was expressed using the upper-tail ratio for the region above two standard deviation units of the mean of the lower-scoring group (*TR*_2.00SD_; see Voracek et al., 2013).

Between-study heterogeneity was assessed using 95% prediction intervals (PIs) and *I*^2^, representing estimates of the dispersion of true effects and the proportion of observed variation attributable to true variation rather than sampling error, respectively (Borenstein et al., 2017). The robustness of the summary effect was examined using influence diagnostics (i.e., standardized residuals, difference in fits (DFFITS) values, Cook’s distances, covariate ratios, hat values, and weights (Viechtbauer & Cheung, 2010)) and leave-one-out sensitivity analyses. Cohen’s *d* effect sizes were interpreted according to the benchmarks proposed by Funder and Ozer (2019), with absolute *d*s = 0.10, 0.20, 0.41, 0.63, and 0.87 representing the bottom thresholds of very small, small, medium, large, and very large effects, respectively.

#### 2.4.1. Subgroup Analyses

To examine potential influences of categorical moderators, we conducted a series of preregistered subgroup analyses. Effect sizes were grouped according to potentially relevant study and sample characteristics, including (i) WLT response format (multiple-choice vs. water-level drawing vs. water-level adjusting), (ii) test setting (group vs. individual administration), (iii) publication status (published vs. unpublished), (iv) the presence of a horizontal reference line (provided vs. not provided), (v) test mode (power vs. speed test), (vi) sex of study’s first author (male vs. female), (vii) vessel shape (rectangular vs. bottle vs. Florence flask vs. jar vs. cylindrical vs. mixed shapes), (viii) stimulus presentation mode (drawing vs. screen vs. real vessel), (ix) continent of data collection (North America vs. Europe vs. Asia vs. Africa vs. Australia), and (x) sample type (pupils vs. undergraduate students vs. general population vs. control group in a clinical study vs. other). Between-group differences were assessed by means of mixed-effects models whereby within-subgroup effects were estimated using random-effects modeling and between-subgroup comparisons using fixed-effect modeling.

#### 2.4.2. Meta-Regression

Effects of continuous moderators were assessed using preregistered precision-weighted linear meta-regressions. First, we examined the influence of publication year on observed effect sizes, which were further evaluated using a cumulative meta-analysis according to publication year. Second, sex effects were regressed on participants’ mean ages. Third, we assessed the influence of participants’ assumed mean birth years (i.e., participants’ mean ages subtracted from studies’ data collection years or, when the data collection years was not indicated, from the publication years) to examine potential cohort effects. Finally, effect sizes were modeled as a function of the number of administered WLT items. Moreover, we used study-level risk-of-bias assessments by means of the scores of the Newcastle–Ottawa Scale for cross-sectional studies (NOS-xs; Carra et al., 2025) to predict effect sizes in an exploratory single weighted meta-regression as a sensitivity analysis.

#### 2.4.3. Dissemination Bias

Considering the limitations of any individual method for detecting dissemination, it has been recommended to triangulate dissemination bias analyses by using both standard and modern bias-detection methods (Siegel et al., 2022). For these preregistered analyses, we closely followed the approach outlined by Oberleiter and Pietschnig (2023). Accordingly, we applied ten complementary methods to examine potential dissemination bias, including *p*-curve (Simonsohn et al., 2014), *p*-uniform (van Assen et al., 2015), *p*-uniform* (van Aert & van Assen, 2026), the power-enhanced funnel plot (Kossmeier et al., 2020), Sterne and Egger’s regression model (Sterne & Egger, 2005), the rank-correlation test (Begg & Mazumdar, 1994), PET-PEESE (Stanley & Doucouliagos, 2014), trim-and-fill (Duval & Tweedie, 2000), the test of excess significance (Ioannidis & Trikalinos, 2007), and selection models (Vevea & Woods, 2005). Technical descriptions of these detection methods are detailed in the online Supplementary Materials (https://osf.io/6fwsa/files/ndfpt (accessed on 28 August 2026)).

#### 2.4.4. Multiverse and Specification-Curve Analyses

To assess the combined influence of categorical moderators, their interactions, and different analytic approaches, we used preregistered multiverse, specification-curve (Voracek et al., 2019), and combinatorial meta-analyses (Olkin et al., 2012). To this end, we assessed different reasonable ways in terms of which data to include (“Which” factors, or the data multiverse) and how to meta-analytically synthesize them (“How” factors, or the model multiverse), with all of these assembled and tested combinatorially (Voracek et al., 2019), thus enabling us to systematically and exhaustively assess potential effects of different specifications as a consequence of researcher degrees of freedom (Oberleiter & Pietschnig, 2023).

Specifically, nine data-multiverse factors were considered, including first author’s sex (male vs. female vs. any [i.e., these two groups combined]), age group (children vs. adolescents vs. adults vs. any), sample type (undergraduates vs. other vs. any), participant origin (North America vs. other vs. any), response mode (draw water level vs. other vs. any), publication status (published vs. not vs. any), test setting (individual vs. group vs. any), presence of a horizontal reference line (yes vs. no vs. any), and vessel shape (rectangular vs. other vs. any). Moreover, one model multiverse factor was used, namely the meta-analytic modeling approach (fixed-effect vs. REML random-effects vs. DerSimonian–Laird random-effects vs. Hunter–Schmidt-typed modeling).

Combinatorial assembly of the nine data-multiverse factors (3 × 4 × 3 × 3 × 3 × 3 × 3 × 3 × 3 = 26,244 specifications) with one model multiverse factor (4 specifications) would yield a total of 104,976 possible reasonable specifications. Because this number is more than 600 times larger than the corpus of primary studies eligible for this meta-analysis, most of the cells (i.e., specifications) in this high-dimensional space were necessarily either empty or populated by only a single case. These empty or single-case specifications were excluded because two or more studies are necessary to calculate a summary effect. Here, 13,204 specifications (12.7% of total) contained at least two independent effect sizes, thus allowing for the calculation of a meta-analytic summary effect.

As an inferential statistical test of the descriptive specification curve against an under-the-null scenario (i.e., if the true effect were indeed null), parametric bootstrapping was used (Voracek et al., 2019). Assuming a true zero effect and treating all study characteristics as fixed, 1000 datasets were generated by randomly drawing effect sizes from normal distributions with an expected value of zero and a standard deviation corresponding to the observed standard error of the respective study. For each generated null dataset, a specification curve was computed. From the resulting 1000 bootstrapped specification curves, the respective 2.5% and 97.5% quantiles were determined to obtain the lower and upper bounds of this randomly generated null distribution. Observed summary effects exceeding these bounds were interpreted as evidence for a non-null effect.

#### 2.4.5. Combinatorial Meta-Analysis

Relevant analytic specifications may remain undetected if certain effect moderators impacting a research literature have not yet been conceptually identified (see Oberleiter & Pietschnig, 2023; Voracek et al., 2019). Therefore, multiverse meta-analytic approaches can be augmented further with combinatorial meta-analysis (Olkin et al., 2012).

This brute-force computational approach estimates summary effects for all possible subsets of studies in a fixed-effect meta-analysis, rather than restricting analyses to theoretically reasonable specifications. Furthermore, this approach allows for the exploratory detection of potential moderators and influential studies based on the distributional characteristics of (all-subset) meta-analytic summary effects alongside corresponding cross-study effect heterogeneity measures, as displayed in GOSH (graphical display of study heterogeneity) plots (Olkin et al., 2012).

However, calculating all the 2^k^ – 1 possible subsets comprising the combinatorial meta-analysis is computationally intractable in the present case (*k* = 267 samples), because, due to combinatorial explosion, this would amount to about 10^78^ distinct models. For practical and feasibility reasons, 100,000 samples from this vast model space were therefore drawn randomly. To account for potential influences of outliers, a stratified sampling procedure based on subset size was applied, thus oversampling the least frequent (i.e., smallest and largest) subset sizes. The resulting GOSH plot was visually inspected to ascertain any patterns indicative of systematic unobserved heterogeneity, such as distinct sub-clusters or multimodal distributions of summary effects.

All statistical analyses were performed using R version 4.3.3 (R Core Team, 2024) with the package metafor (Viechtbauer, 2010). To illustrate the geographic distribution of samples, R code from Oberleiter et al. (2023) was adapted (https://osf.io/3qjrd/files/kax59 (accessed on 28 August 2026)). Analysis code for the multiverse/specification-curve and combinatorial meta-analyses followed the implementation of Voracek et al. (2019); see https://osf.io/nkv46 (accessed on 28 August 2026). The R code for the present analyses is available from https://osf.io/6fwsa/files/osfstorage (accessed on 28 August 2026). Dissemination bias detection analyses were conducted using the MetaShine web application (Siegel et al., 2021; https://the-meta-analysis-project.shinyapps.io/MetaShine/ (accessed on 28 August 2026)).

## 3. Results

### 3.1. Final Meta-Analytic Sample

In all, effect sizes from 89 studies either included in Pavlovic et al. (2008), Pietschnig and Voracek (2011), or both, and 77 additionally identified studies were included in the present meta-analysis (see online Supplementary Figure S5.1 for an overview of included samples by publication year; https://osf.io/6fwsa/files/ndfpt (accessed on 28 August 2026); references of all included studies are available from https://osf.io/6fwsa/files/udpvn (accessed on 28 August 2026)). The final dataset comprised effect sizes from 267 independent samples (*N* = 38,314), stemming from 166 studies (43 effects, or 16.1%, originated from unpublished samples) from the period 1964 to 2025. Participant mean age was 18.6 years (*Mdn* = 17.8, *SD* = 11.1, *IQR* = 11.3–21.4, and range: 5–79 years) and, based on samples’ reported participant mean ages, assumed (mean) birth years of participants across studies ranged from 1901 to 2018. Most participants were from North America (62%), followed by Europe (25%), Africa (5%), Asia (3%), and Australia (1%; see online Supplementary Figure S5.2 for an illustration of sample origin, at https://osf.io/6fwsa/files/ndfpt (accessed on 28 August 2026)). The majority of samples consisted of undergraduate college students (*k* = 110) and elementary or secondary school pupils (*k* = 99), while others were based on the general population (*k* = 16), control groups from clinical studies (*k* = 4), or other specific groups (*k* = 31), such as STEM experts (Tomai et al., 2024), athletes (Meneghetti et al., 2021), or parents (Walker et al., 1981). Across the included studies, 93% of calculated effect sizes were positive (*d* > 0), thus indicating a male advantage in WLT performance (*d* range: −0.56 to 1.84). Characteristics of the included studies are detailed in Table 1 (the full dataset is provided at https://osf.io/6fwsa/files/me6uy (accessed on 28 August 2026)).

### 3.2. Main Analysis

A precision-weighted REML random-effects meta-analysis of sex differences in WLT performance yielded a summary effect of *d* = 0.501 (95% CI [0.465, 0.537], 95% PI [0.120, 0.882], *p* < .001) or Hedges *g* = 0.497 (95% CI [0.461, 0.533], 95% PI [0.118, 0.876], *p* < .001), respectively (see online Supplementary Figure S5.3 for a forest plot; https://osf.io/6fwsa/files/ndfpt (accessed on 28 August 2026)), indicating a moderate-to-large effect. This summary effect corresponds to approximately 33% nonoverlap between the assumed male and female performance distributions (Cohen’s *U*_1_ = 0.33, 95% CI [0.31, 0.35]) and, under the assumption of distributional normality and equal variances, almost three times as many men as women in the performance region above two standard deviations rightwards to the female mean (*TR*_2.00SD_ = 2.94). Because variances of men and women differed substantially in some cases, an exploratory sensitivity analysis based on the effect size e, a standardized mean difference estimate that does not assume homogeneity of variances (Aoki, 2020), was conducted on a subset of studies that reported numerical values for means, *SD*s and sample sizes within subgroups of men and women. Results were virtually identical to those obtained using Cohen’s *d* (for numerical details, see Supplementary Table S6.1; https://osf.io/6fwsa/files/ndfpt (accessed on 28 August 2026)). Moreover, a multilevel model using a restricted maximum likelihood estimator with (participant-wise non-overlapping) samples nested within studies yielded virtually identical estimates (*d* = 0.498; 96% CI [0.458, 0.538], *p* < .001). Consequently, we based our subsequent analyses on the full dataset using the *d* metric in a two-level approach.

Between-study effect heterogeneity, albeit moderately sized, was significant (*Q*(266) = 593.97, *p* < .001, *I*^2^ = 52.9, τ = 0.19), thus suggesting the presence of further, unobserved effect moderators. Outlier diagnostics using influence statistics (see online Supplementary Figure S5.4; https://osf.io/6fwsa/files/ndfpt (accessed on 28 August 2026)) and a leave-one-out sensitivity analysis yielded three effect sizes as potential leverage points. However, re-estimating the models after excluding these cases resulted in almost no change in the summary effect (Δ*d* < 0.03). Accordingly, all subsequent analyses were conducted using the full dataset.

### 3.3. Effect-Moderator Analyses

#### 3.3.1. Subgroup Analyses

To assess whether effect sizes varied as a function of test and sample characteristics, a series of preregistered mixed-effects subgroup analyses was conducted (for the corresponding subgroup summary effects, see Table 2).

Across all examined data subsets, effect sizes invariably were positive, once again indicating sex differences with better performances in men compared to women. These effects were consistent across differences in response format (multiple-choice vs. water-level drawing vs. water-level adjusting; *Q*(2) = 3.82, *p* = .15), test setting (group vs. individual test; *Q*(1) = 0.01, *p* = .92), publication status (*Q*(1) = 0.58, *p* = .45), presence of a horizontal reference line (*Q*(1) = 1.06, *p* = .30), presence of a time limit (power vs. speed test; *Q*(1) = 0.03, *p* = .87), first author’s sex (*Q*(1) = 0.03, *p* = .86), and stimulus presentation mode (drawn on paper vs. depicted on screen vs. with photograph of a vessel vs. with model of a real vessel; *Q*(3) = 0.87, *p* = .83).

Effect sizes varied significantly as a function of vessel shape (rectangular vs. bottle vs. Florence flask vs. jar vs. cylindrical vs. mixed shapes; *Q*(5) = 13.65, *p* = .018). Specifically, exploratory post-hoc pairwise comparisons indicated that this effect moderation was exclusively driven by the mixed-shape category, yielding numerically larger sex differences compared to some single-shape categories (rectangles: *Q*(1) = 6.41, *p* = .011; bottles: *Q*(1) = 6.94, *p* = .008; Florence flasks: *Q*(1) = 5.23, *p* = .022; jars: *Q*(1) = 9.25, *p* = .002). All other pairwise comparisons between vessel shapes yielded no significant differences (all *Q*s(1) ≦ 2.63, all *p*s ≧ .11). We note that Bonferroni-adjustments of *p*-values would yield no significant differences in any of the post-hoc subtests of vessel shape.

The observed meta-analytic summary effect differed significantly across age groups (children vs. adolescents vs. adults; *Q*(2) = 24.81, *p* < .001). Exploratory post-hoc pairwise comparisons indicated that observed male advantages were larger among adults than among children or adolescents (*Q*(1) = 20.83, *p* < .001 and *Q*(1) = 12.24, *p* < .001, respectively, both of which would have survived Bonferroni adjustment), whereas no significant difference was observed between children and adolescents (*Q*(1) = 0.44, *p* = .51). Beyond these age-related differences, sex differences generalized across sample compositions. Moderate-to-large effect sizes favoring men were observed irrespective of the continent where the data were collected (North America vs. Europe vs. Asia vs. Africa vs. Australia; *Q*(4) = 6.36, *p* = .17) and sample type (undergraduate students vs. pupils vs. general population vs. control group in a clinical study vs. other; *Q*(4) = 6.09, *p* = .19).

#### 3.3.2. Meta-Regressions

To assess continuous moderators, several preregistered precision-weighted linear meta-regression models were calculated (see online Supplementary Table S7.1; https://osf.io/6fwsa/files/ndfpt (accessed on 28 August 2026)).

The number of test items administered did not significantly predict sex differences in WLT performance (*k* = 259, *b* = 0.001, 95% CI [−0.001, 0.003], *R*^2^ < 0.1% of between-study heterogeneity accounted for, *p* = .41), indicating that effect strength was not associated with test length.

In contrast, mean participant age positively predicted the strength of observed sex differences (*k* = 216, *b* = 0.007, 95% CI [0.003, 0.011], *R*^2^ = 15.2%, *p* < .001), indicating increasing male advantages with age. However, the data comprised comparatively few older samples (*Mdn* age = 17.8 years, *IQR*: 11.3–21.4), with a median age of 21.4 years within the adult participant group (*IQR*: 19.7–25.5). Therefore, to explore the potential influence of atypical observations, the 20% most atypical cases were identified based on Mahalanobis distances. Re-estimating the model after excluding these cases yielded a slightly larger slope compared to the full model (*k* = 182, *b* = 0.009, 95% CI [0.003, 0.015], *R*^2^ = 15.2%, *p* = .003), thus indicating that the age effect was salient and not driven by multivariate outliers (see Figure 2).

Moreover, publication year was negatively related to effect strength (*k* = 267, *b* = −0.005, 95% CI [−0.008, 0.003], *R*^2^ = 8.1%, *p* < .001), indicating cross-temporally declining effect sizes. Visual inspection of the cumulative forest plot according to publication year was consistent with this pattern (see online Supplementary Figure S5.5; https://osf.io/6fwsa/files/ndfpt (accessed on 28 August 2026)). Exploratory analyses indicated that the association between publication year and effect strength was substantially attenuated, but remained statistically significant, after controlling for mean participant age (*k* = 216, *b* = −0.003, 95% CI [−0.006, 0.001], *R*^2^ = 16.1%, *p* = .021). Additionally, an exploratory linear regression indicated that mean participant age was negatively, albeit non-significantly, predicted by publication year (*b* = −0.051 (95% CI [−0.153, 0.051], *R*^2^ = 0.4%, *p* = .33).

To explore whether this cross-temporal decline varied by participant age, a meta-regression including the interaction between publication year and samples’ participant mean ages was conducted. The interaction term was positive and statistically significant (*k* = 216, *b* = 0.0003, 95% CI [0.0001, 0.006], *R*^2^ = 20.7%, *p* = .005), indicating stronger cross-temporal effect declines in younger participants compared to older test-takers. To further investigate this pattern, separate exploratory meta-regressions of effect sizes on publication year were estimated for each age group. These analyses indicated that effect declines were stronger among adolescents (*k* = 56, *b* = −0.011, 95% CI [−0.017, 0.004], *R*^2^ = 27.6%, *p* = .001; see Figure 3) than among adults (*k* = 152, *b* = −0.004, 95% CI [−0.006, −0.001], *R*^2^ = 10.8%, *p* = .020) and children, whereas the latter were not statistically significant (*k* = 52, *b* = −0.004, 95% CI [−0.010, 0.001], *R*^2^ < 0.1%, *p* = .12). Follow-up interaction models, testing pairwise differences in the slopes of the cross-temporal trends across age groups showed that the effect decline was significantly steeper in adolescents than in adults (*b* = 0.007, 95% CI [0.001, 0.014], *p* = .033), while the slope differences between children and adolescents and between children and adults did not reach nominal significance (*b*s = −0.007, 95% CI [−0.014, 0.001], and 0.001, 95% CI [−0.001, 0.001]; *p*s = 0.10 and 0.75, respectively).

Consistent with this pattern, a meta-regression of predicting effect sizes based on the assumed mean birth year of study participants yielded a statistically significant negative effect (*k* = 216, *b* = −0.005, 95% CI [−0.007, −0.002], 10.0%, *p* < .001), suggesting smaller sex differences in more recent birth cohorts. Exploratory analyses further indicated that this association was attenuated, but remained statistically significant and numerically non-trivial, after controlling for mean participant age (*k* = 216, *b* = −0.003, 95% CI [−0.001, −0.009], 15.4%, *p* = .040). An exploratory sensitivity analysis restricted to studies explicitly reporting the year of data collection yielded a numerically virtually identical moderating effect of birth year compared to the full dataset, although the effect failed to reach nominal statistical significance (*k* = 34, *b* = −0.005, 95% CI [−0.012, 0.002], *p* = .132).

Finally, in an exploratory meta-regression examining influences of study-level risk of bias, larger study-level NOS-xs scores were associated with smaller effect sizes (*k* = 267, *b* = −0.039, 95% CI [−0.072, −0.005], *p* = .024, *R*^2^ < 0.01), thus indicating smaller effect sizes of studies with lower risk of bias.

### 3.4. Dissemination Bias

No evidence for dissemination bias was detected in any of the used methods. No method indicated that confounding dissemination bias materially influenced the observed summary effect according to the criteria described by Siegel et al. (2022). The numerical results of these bias analyses are detailed in the online Supplementary Materials (Supplementary Table S4.1 and Supplementary Figures S4.1 and S4.2; https://osf.io/6fwsa/files/ndfpt (accessed on 28 August 2026)).

### 3.5. Multiverse Meta-Analyses

#### 3.5.1. Specification-Curve Meta-Analyses

Across the multiverse of specifications, almost all combinations yielded a positive summary effect, indicating sex differences favoring men (see Figure 4). More specifically, 12,438 out of the 13,204 summary effects (i.e., 94%) were significantly positive (*p* < .05) and not a single specification yielded a significant negative effect. Effect sizes ranged from *d* = −0.25 to 1.34 (mean *d* = 0.49, *Mdn d* = 0.50, *IQR*: 0.40–0.57). Estimates near the center tended to be more precise (i.e., associated with narrower confidence intervals) compared to effects at the lower and upper ends of the effect size distribution. Furthermore, the inferential meta-analytic specification curve deviated substantially from the under-the-null scenario (see Figure 5). In all, these findings underline the remarkable generality of sex differences in WLT performance.

The *p*-value distribution for the summary effects of all meta-analytic specifications is provided in the online Supplementary Materials (Supplementary Figure S5.6; https://osf.io/6fwsa/files/ndfpt (accessed on 28 August 2026)).

#### 3.5.2. Combinatorial Meta-Analysis

Visual inspection of the GOSH plot was consistent with the findings from the specification-curve analyses (see Figure 6). Specifically, across all examined subsets (100,000 randomly drawn), summary effects averaged *d* = 0.49 (*Mdn* = 0.48, *IQR*: 0.47–0.50). Between-study heterogeneity was small to moderate across most subsets, averaging *I*^2^ = 52.5 (*Mdn* = 55.1, *IQR*: 49.4–57.9). Subsets including at least one of the three previously identified leverage points (i.e., De Avila & Havassy, 1974; Exp. 5 of Harris et al., 1977; and Control group 1 of S. A. Sorby & Veurink, 2019) showed increased between-study heterogeneity, but not meaningful deviations in either summary effects or heterogeneity estimates compared with the remaining subsets, thus further supporting the robustness and generality of the observed sex differences.

## 4. Discussion

This preregistered meta-analysis provides evidence for remarkably general, moderate-to-large sex differences in WLT performance favoring men. This effect generalized across the majority of examined sample characteristics, administered WLT formats, and analytic approaches. Virtually identical observations emerged based on standard (REML random-effects), specification-curve, and combinatorial meta-analyses. Further, neither stimulus presentation modality nor test setting (i.e., individual vs. group testing) meaningfully altered the effect strength, indicating robustness of the observed sex differences across contextual variations in test administration. Effect sizes were differentiated according to participant age, with larger sex differences seen among older participants; also, effect sizes declined over historical time. These findings are consistent with older, less comprehensive accounts documenting moderate male advantages compared to women in WLT performance (Pavlovic et al., 2008; Pietschnig & Voracek, 2011; Voyer et al., 1995). These results present several points of interest, as discussed below.

### 4.1. Participant Age as Effect Moderator

The strongest moderating influence observed in this meta-analysis pertained to mean participant age. These effects appeared to be particularly robust, remaining observable even after adjusting our analyses for potential multivariate outliers. These findings support previous findings, indicating larger sex differences among older participants (Linn & Petersen, 1985; Pietschnig & Voracek, 2011; Voyer et al., 1995).

Specifically, sex differences were somewhat smaller in childhood (*d* = 0.36), whereas larger in adolescence and adulthood in particular (*d* = 0.41 and *d* = 0.57, respectively). In terms of psychological development, such differences in effect strength may be interpreted as evidence for the “bent twig” hypothesis (Robert & Harel, 1996; Vasta & Liben, 1996). According to this idea, perinatal and postnatal endocrine and neurobiological mechanisms may give rise to sex-specific predispositions for certain preferences and abilities, which, in turn, are reinforced through psychosocial processes and differential experiences as individuals mature (Halpern, 2013).

With regard to WLT performance in particular (and spatial ability more broadly), boys, on average, may show a stronger tendency than girls to engage in spatial activities at an early age (Ceci et al., 2009; Todd et al., 2018). These tendencies may be further reinforced through environmental influences, such as gender-differentiated toy play that may be explicitly or implicitly encouraged by parents (Liben et al., 2018; Kollmayer et al., 2018b). Retrospective reports from adults indicate that individuals who preferred nonspatial toys and activities during childhood exhibit lower WLT performance (Doyle et al., 2012) and thus contribute to larger WLT-based sex differences (Voyer et al., 2000) than individuals who preferred spatial forms of play, thus tentatively supporting this interpretation.

As children enter school age, sex-related stereotypes concerning interest and performance in different subjects (e.g., languages and arts for girls and science and mathematics for boys) communicated within educational contexts may further reinforce differential engagement in spatial activities of boys and girls (Kollmayer et al., 2018a). These processes may contribute to differences in spatial self-concepts (Signorella & Liben, 2024), spatial confidence (Nardi et al., 2013), and spatial anxiety (Geer et al., 2024) observed in adolescents and young adults, which may further amplify differences in spatial engagement and performance. Alternatively, differential spatial engagement between both sexes could be promoted by supportive environments that allow a more stringent pursuit of individual interests (Ceci & Williams, 2011; Halpern et al., 2007; for a discussion about parallel ideas concerning sex differences in reading and mathematics performance, see Giofre et al., 2020; Oberleiter et al., 2023).

### 4.2. Cross-Temporal Effect-Size Changes

Precision-weighted meta-regressions indicated that effect sizes were negatively related to both publication year and mean participant birth year, indicating a decline in the strength of sex differences over time. These findings are consistent with previous evidence of negative birth-cohort associations (Voyer et al., 1995). Smaller sex differences in more recent birth cohorts may reflect a consequence of changes in sex-related societal attitudes and educational practices.

In this context, previous research has highlighted the role of educational interventions in shaping more egalitarian gender role attitudes in adolescents across cultural contexts (Dhar et al., 2022; Du et al., 2021; Kågesten et al., 2016). Moreover, more egalitarian gender role attitudes in adolescence have been linked to smaller sex differences in mathematics performance (Taraszow et al., 2024), which, in turn, is moderately associated with spatial skills (Atit et al., 2022). Consistent with this interpretation, the present analyses yielded larger effect declines among adolescents than among adults and children. This pattern suggests that changes in educational socialization practices may have become less differentiated across cohorts, thereby contributing to the attenuation of sex differences in spatial perception over recent decades. It is possible that changes in educational practices, gender norms, or availability of targeted spatial-skill training may be responsible for at least a certain extent of the observed trajectory.

At the same time, the findings indicate a negative association between primary study publication year and sex-effect strength, thus contrasting past conclusions of cross-temporal effect size stability (Pietschnig & Voracek, 2011). One possible interpretation is that the observed pattern reflects a so-called inflated decline effect (Protzko & Schooler, 2017); namely, it mirrors more general evidence for effect sizes apparently decreasing over time due to publication bias-related artifacts (Pietschnig et al., 2019). However, in the present meta-analysis, none of the various dissemination-bias detection methods applied provided evidence for publication bias, thus rendering this interpretation tentative.

Alternatively, the negative association between publication year and effect size may partly reflect the link between participant birth year and effect strength. Although this association did not reach nominal statistical significance when restricting the analysis to studies explicitly reporting data collection years, the strength of the estimated moderator effect was virtually identical to that obtained in the full dataset, supporting the interpretation of declining sex differences across cohorts. Moreover, the association between birth year and effect strength appeared to be attenuated, but not eliminated, after controlling for mean participant age. This suggests that the observed decline may partly reflect differences in participant age across publication periods, with a tendency for more recently published studies to include younger samples.

In all, our findings suggest that the observed cross-temporal decline possibly reflects a genuine cohort-related change. However, because the possible explanations discussed above cannot be fully disentangled based on the available database, it cannot be ruled out that both cohort effects and inflated decline effects contribute to the attenuation of sex differences over time. Further research is therefore needed to clarify the mechanisms underlying these cross-temporal changes.

### 4.3. Between-Studies Heterogeneity

Despite our observation that sex differences generalized over most moderators, between-studies heterogeneity was non-trivial (average *I*^2^ = 52.5 in combinatorial estimations). This is not uncommon in meta-analyses, with median *I*^2^ estimates of meta-analyses in the psychological literature ranging from 71% to 74% (van Erp et al., 2017; Stanley et al., 2018). However, additional sources of variability may have yet to be identified to account for the residual heterogeneity.

Such additional sources may be conceivably related to sociocultural influences in which spatial skills develop. Some cross-cultural studies have reported larger sex differences in spatial abilities in more gender egalitarian countries (Janssen & Geiser, 2012; Lippa et al., 2010). Furthermore, observations of moderate-to-large associations between mathematical and spatial skills (Atit et al., 2022; Xie et al., 2020) and larger sex differences in STEM performance in more prosperous countries (Oberleiter et al., 2023) may mean that macro-indicators of national equality or prosperity may account for some variability in sex differences.

### 4.4. Implications for STEM Achievement

The results of the multiverse/specification-curve and combinatorial meta-analyses provide strong evidence for the generality of medium-to-large sex differences (favoring men) in WLT performance. The observed generalization across procedural and analytic specifications clearly indicates a male advantage in spatial perception (i.e., extrinsic-static spatial skills; Linn & Petersen, 1985; Newcombe & Shipley, 2014).

Sex differences in spatial ability have been frequently discussed as one of many factors explaining the underrepresentation of women in scientific fields (Ceci et al., 2009; Reilly et al., 2017). A substantial body of research indicates that spatial abilities are associated with mathematics performance (see Atit et al., 2022; Xie et al., 2020, for meta-analyses) and STEM-related educational pathways (Wai et al., 2009; Webb et al., 2007), including studies specifically employing WLTs (Scott et al., 2023; Voyer, 1998). The current results add to this literature by showing that small, but reliable, sex differences in WLT performance are already observable in childhood, an age period in which spatial abilities have repeatedly been shown to predict later STEM-related outcomes (Frick, 2019; Gilligan et al., 2017; Wolfgang et al., 2001).

Moreover, the presently observed summary effect of *d* = 0.50 corresponds to almost three times as many men as women in the upper distributional tail beyond two standard deviations above the female mean, assuming normality of distributions and equality of variances. Such imbalances at the highest ability levels may be one of several factors contributing to the smaller proportion of women observed in STEM occupations (Eurostat, 2026; Signorella & Liben, 2024). However, it needs to be acknowledged that most of the present data were collected in North America and Western Europe, thus limiting the generalizability of this interpretation.

The observed sex effect increased with age, which may point to spatial-ability training as a potential avenue for attenuating sex differences in spatial perception. In this vein, training studies have shown that performance in a wide range of spatial tasks including WLTs can be improved through targeted interventions and experience, with men and women responding equally well to spatial training (Uttal et al., 2013a). Moreover, spatial interventions have been linked to improvements in STEM-related attainment and achievement (Stieff & Uttal, 2015; see Hawes et al., 2022, for a meta-analysis), with some studies pointing to particularly strong effects among girls and women (S. Sorby et al., 2018; S. A. Sorby & Veurink, 2019). It has therefore been proposed that implementing spatial training during early developmental stages may help to reduce later emerging sex effects in spatial cognition and STEM performance (Reilly et al., 2017). Consequently, these findings have led to calls for the integration of spatial training into educational practice (Gilligan-Lee et al., 2022; Newcombe & Frick, 2010). Nevertheless, it remains unclear whether training-induced improvements in WLT performance may eventually yield downstream gains in STEM-related outcomes. Moreover, longitudinal evidence is needed to clarify whether such gains may ultimately contribute to increasing the representation of women in STEM disciplines.

### 4.5. Limitations

Several limitations of this meta-analysis, inherited from the primary literature it synthesizes, are evident. First, none of the included studies formally tested measurement invariance between male and female participants, which is required to ensure fair and valid test-score comparisons (Navarro-González et al., 2024). However, evidence from other spatial tasks suggests that potential measurement non-invariance may not substantially compromise the interpretability of sex differences in spatial cognition (Maeda & Yoon, 2016; Xu et al., 2016). Moreover, the observed generality of sex differences across samples, task variants, and analytic specifications suggests that the main findings are unlikely to be solely attributable to differential item functioning.

Second, 242 out of the 267 included samples (91%) originated from WEIRD countries, including 128 from the USA alone. This geographical concentration in all likelihood limits the generalizability of results across cultural contexts. The scarce evidence from non-WEIRD studies is quite mixed. For example, Davis et al. (2021) reported a small female advantage among seminomadic pastoralist-forager children from northern Namibia, whereas Li et al. (1999a, 1999b) observed higher overall performance among Chinese compared to American students, although with male advantages in both groups. However, further research based on measurement-invariant data is required to establish valid cross-cultural comparisons.

Third, substantial between-studies effect heterogeneity remained after accounting for numerous theoretically relevant moderators, suggesting that additional sources of variability in sex differences in WLT performance have yet to be identified. However, our multiverse meta-analyses demonstrated that the observed summary effect was stable across a wide range of reasonable specifications, thus supporting the robustness of the reported sex differences despite remaining residual unexplained variability.

Fourth, sex differences appeared to be smaller in studies that indicated lower risk of bias according to the NOS-xs. However, variance explained was trivial (*R*^2^ < 0.01), thus indicating no meaningful effects of individual study-level bias risk on effect sizes.

Finally, the negative association of birth years with effect sizes needs to be interpreted with caution because this association was attenuated after adjusting for mean participant age. However, publication year did not significantly predict mean age, suggesting that the apparent cohort-related decline cannot be attributed solely to effects of systematically younger participants in more recently investigated birth cohorts.

## 5. Conclusions

This comprehensive meta-analysis provides evidence for medium-to-large sex differences in water level task performance favoring men. This sex effect appears to be remarkably robust and generalizes across a large number of potential moderator variables, but is stronger in older individuals and appears to have been declining over time. In all, our findings indicate that the concept of horizontality invariance may not emerge uniformly across individuals and, in some cases, may remain incompletely developed even in adulthood, thereby challenging an important assumption of Piagetian stage theory.

## Figures and Tables

**Figure 1:**
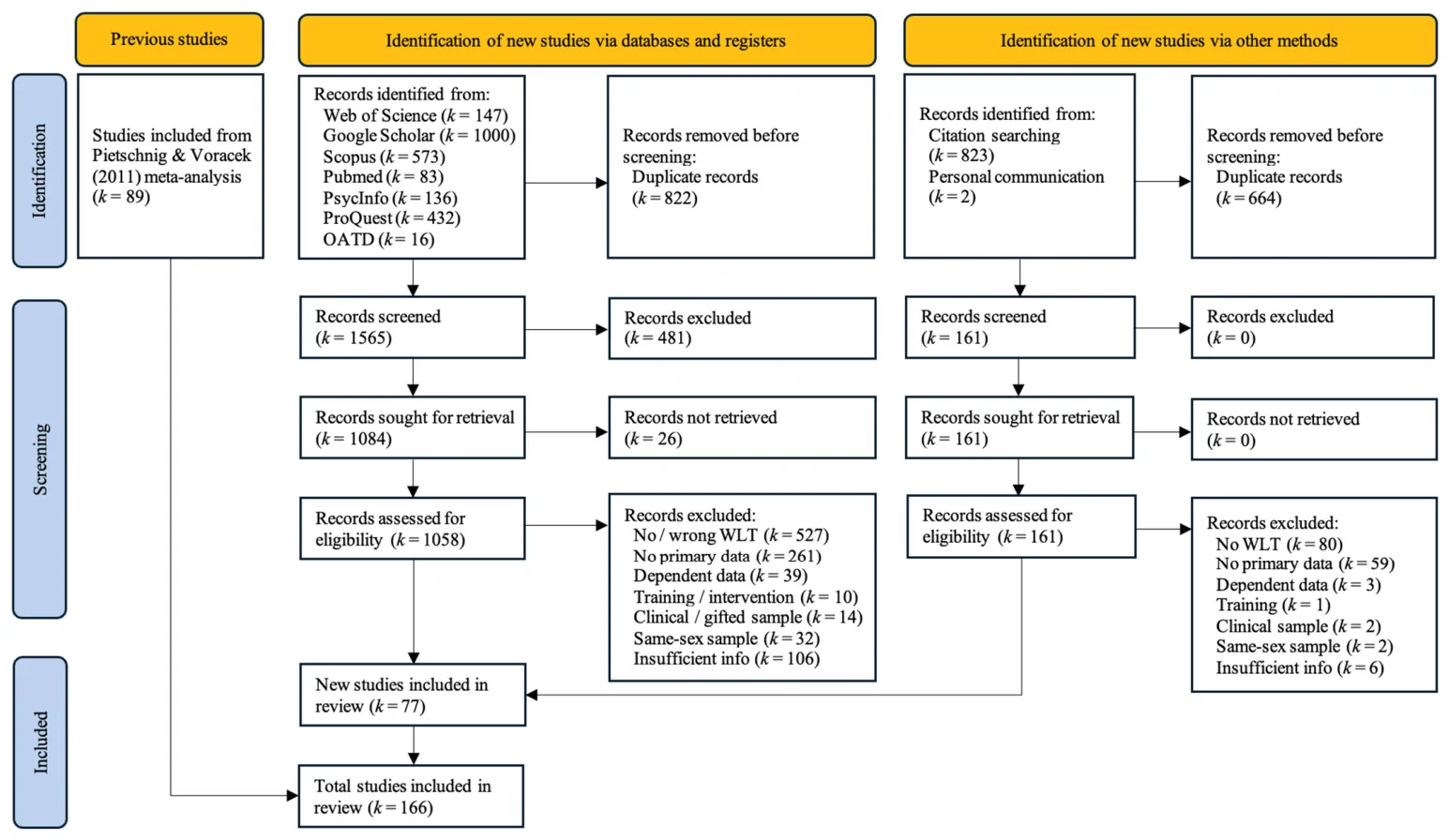
PRISMA 2020 flow diagram (Page et al., 2021; Pietschnig & Voracek, 2011).

**Figure 2:**
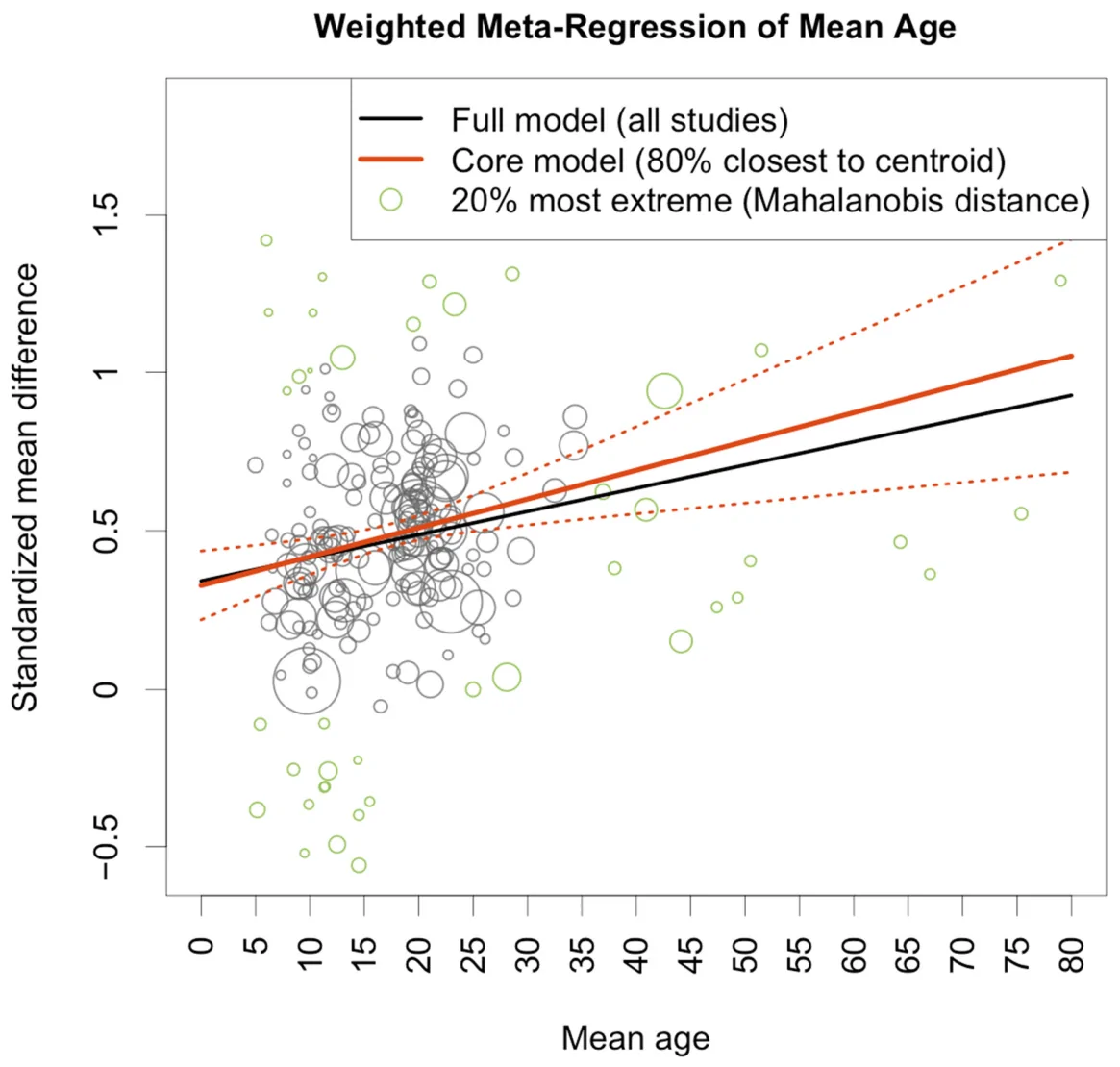
Precision-weighted meta-regression of participant age. Note. The 20% of most extreme cases (i.e., furthest from the cluster centroid) in the joint distribution of mean participant age and sex differences in WLT performance were identified using Mahalanobis distances. The dashed lines represent the 95% confidence interval around the core- model regression line. Larger bubbles represent larger sample sizes (i.e., higher precision).

**Figure 3:**
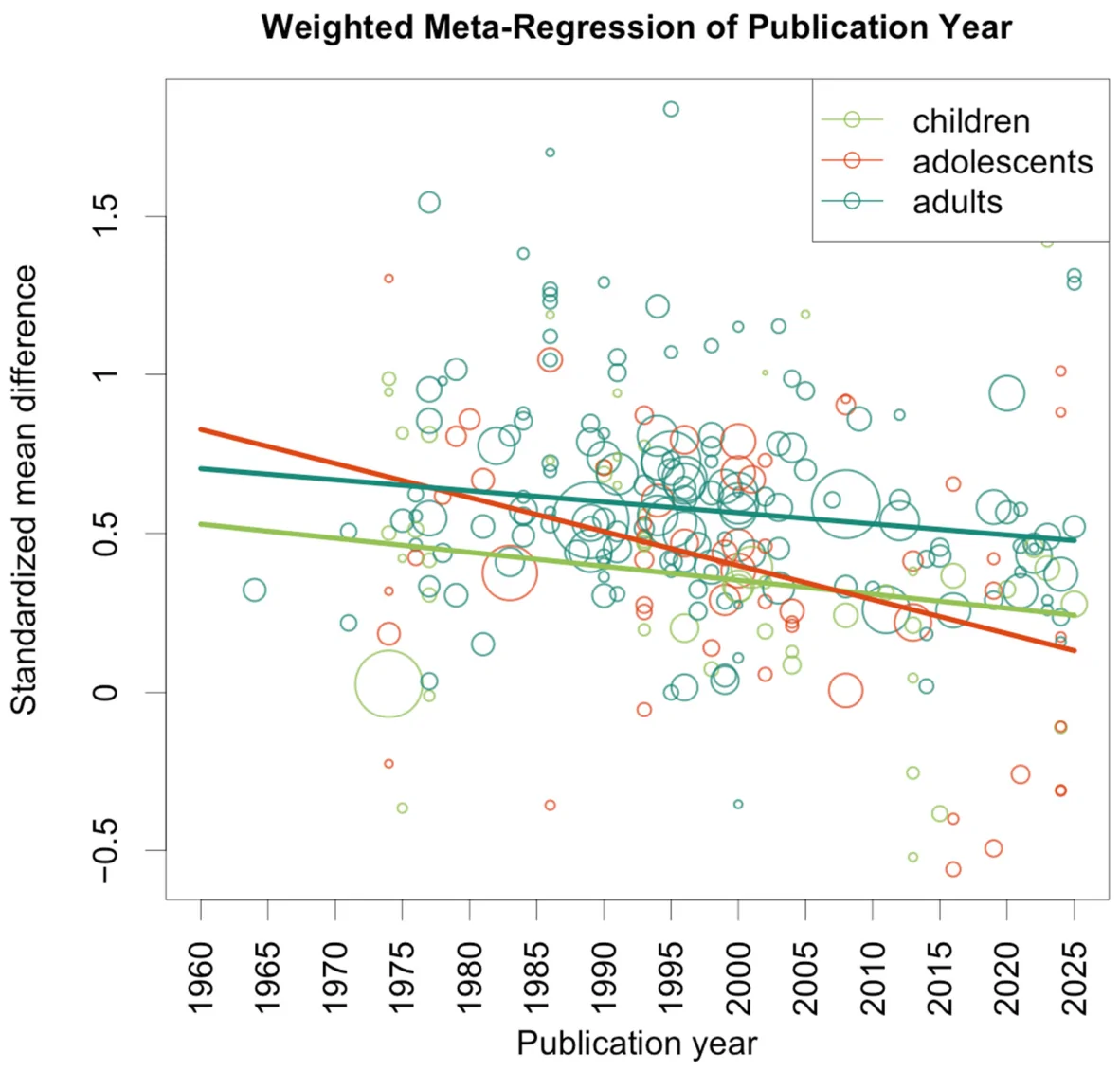
Precision-weighted meta-regression of publication year. Note. Larger bubbles represent larger sample sizes (i.e., higher precision), scaled relative to the maximum sample size across all age groups.

**Figure 4:**
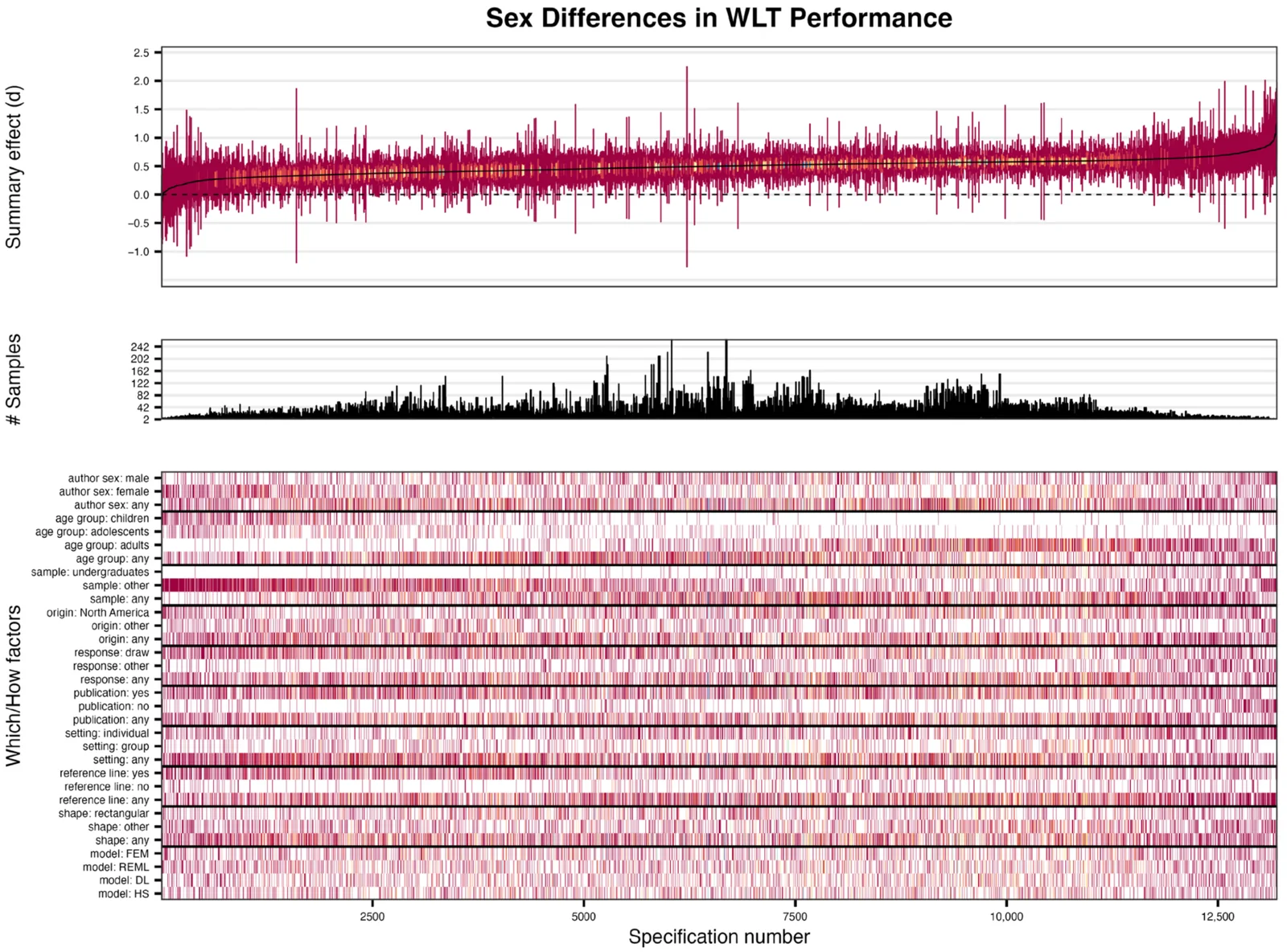
Descriptive meta-analytic specification-curve plot. Note. Meta-analytic summary effects (alongside 95% confidence intervals), ordered by increasing strength, are displayed in the top panel. Confidence intervals intersecting the horizontal dashed reference (of *d* = 0) represent nominally non-significant summary effects. The number of samples represented in each specification is shown in the middle panel. The bottom panel indicates the combination of “Which” (data multiverse) and “How” (model multiverse) factors comprising each specification. Top and bottom panel colors indicate estimates’ precision, with colder vs. warmer colors indicating higher vs. lower precision, respectively.

**Figure 5:**
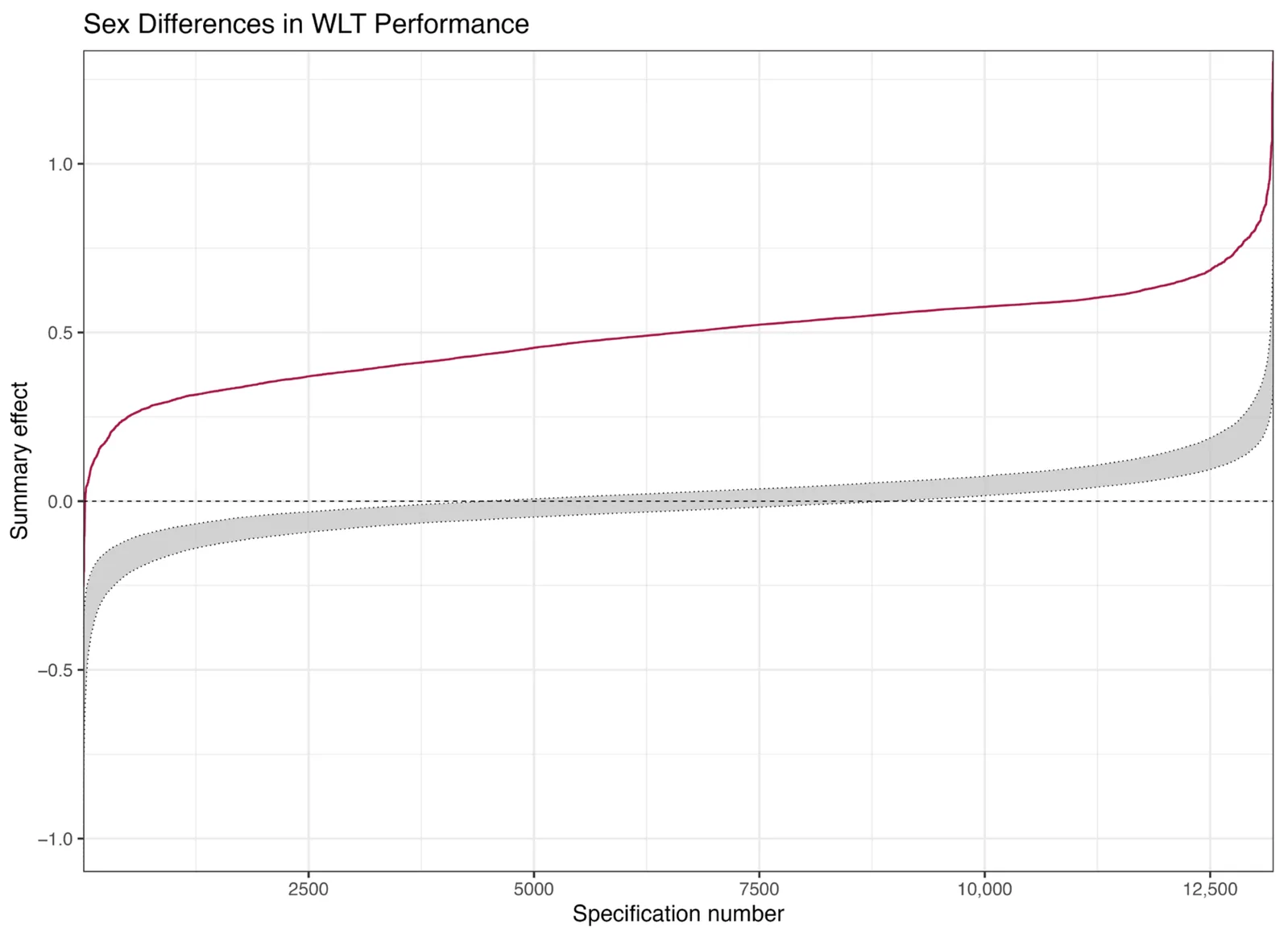
Inferential meta-analytic specification-curve plot. Note. The solid red line illustrates the observed specification curve; the dashed line represents a null effect. The limits of the grey area represent the upper and lower bounds of the 95% confidence interval for each specification, based on 1000 parametrically bootstrapped specification curves simulated under an under-the-null scenario (i.e., true null effect).

**Figure 6:**
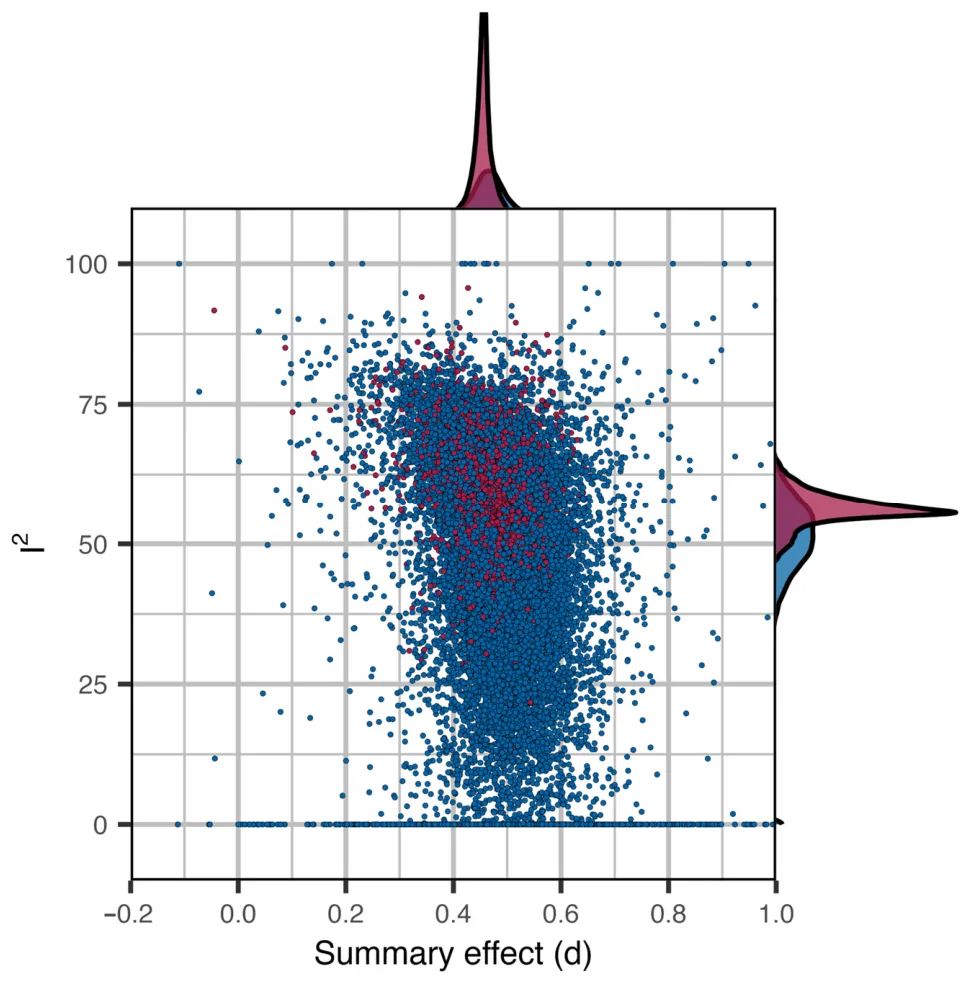
GOSH (graphical display of study heterogeneity) plot for combinatorial meta-analysis. Note. The dots represent the summary effects of 100,000 randomly drawn subsets of all possible meta-analytic combinations. Estimates from meta-analytic subsets including at least one of the three identified leverage points are highlighted in red.

**Table 1:** Characteristics of included studies.

Reference	*N*	Percent Male	Sample Type	Mean Age	Origin	Pub	Source	Test Mode	Items	Shape	Response Format	*d*
Rebelsky (1964)	128	46.09	undergrad	23.00	USA	p	P&V	paper	4	rect	draw WL	0.322
Morris (1971)	64	50.00	undergrad	20.50	USA	p	P&V	paper	4	rect	draw WL	0.507
Morris (1971)	64	50.00	undergrad	20.50	USA	p	P&V	paper	4	rect	draw WL	0.219
De Avila and Havassy (1974)	1180	49.75	pupils	9.72	Canada	p	P&V	paper	17	rect	draw WL	0.026
Liben (1974)	50	54.00	pupils	9.00	USA	p	P&V	paper	4	rect	draw WL	0.501
Liben (1974)	44	45.45	pupils	9.00	USA	p	P&V	paper	4	rect	draw WL	0.987
Mitchelmore (1974)	16	50.00	pupils	9.60	Jamaica	np	Pro- Quest	paper	4	bottle	draw WL	0.944
Mitchelmore (1974)	16	50.00	pupils	11.15	Jamaica	np	Pro- Quest	paper	4	bottle	draw WL	1.305
Mitchelmore (1974)	16	50.00	pupils	12.75	Jamaica	np	Pro- Quest	paper	4	bottle	draw WL	0.319
Mitchelmore (1974)	16	50.00	pupils	14.40	Jamaica	np	Pro- Quest	paper	4	bottle	draw WL	−0.227
Mitchelmore (1974)	127	42.52	pupils	14.50	Jamaica	np	Pro- Quest	paper	4	bottle	draw WL	0.185
Thomas and Jamison (1975)	134	46.27	undergrad	19.42	USA	p	P&V	real vessel	48	rect	adjust WL	0.540
Maxwell et al. (1975)	35	51.43	pupils	8.96	USA	p	Scholar	paper	8	cylindrical	draw WL	0.816
M. S. Miller (1975)	16	50.00	pupils	9.90	Canada	np	Pro- Quest	paper	16	NA	draw WL	0.422
M. S. Miller (1975)	24	50.00	pupils	9.90	Canada	np	Pro- Quest	paper	16	NA	draw WL	−0.367
E. Geiringer (1976)	60	50.00	pupils	36.94	USA	np	P&V	real vessel	24	florence flask	adjust WL	0.624
E. Geiringer (1976)	40	50.00	pupils	64.28	USA	np	P&V	real vessel	24	florence flask	adjust WL	0.465
E. Geiringer (1976)	40	50.00	pupils	75.40	USA	np	P&V	real vessel	24	florence flask	adjust WL	0.554
E. R. Geiringer and Hyde (1976)	60	50.00	pupils	11.00	USA	p	P&V	real vessel	24	florence flask	adjust WL	0.512
E. R. Geiringer and Hyde (1976)	60	50.00	pupils	18.00	USA	p	P&V	real vessel	24	florence flask	adjust WL	0.424
Abravanel and Gingold (1977)	50	52.00	pupils	9.58	USA	p	P&V	paper	5	cylindrical	draw WL	0.307
Abravanel and Gingold (1977)	50	48.00	pupils	9.90	USA	p	P&V	paper	5	bottle	draw WL	0.416
Abravanel and Gingold (1977)	30	46.47	pupils	10.16	USA	p	P&V	paper	5	cylindrical	draw WL	−0.010
Harris et al. (1977)	158	30.38	NA	NA	NA	p	P&V	paper	16	mixed	MC	0.953
Harris et al. (1977)	61	54.10	NA	NA	NA	p	P&V	paper	16	mixed	MC	0.812
Harris et al. (1977)	158	33.54	NA	NA	NA	p	P&V	paper	16	mixed	MC	0.854
Harris et al. (1977)	110	40.91	NA	NA	NA	p	P&V	paper	16	mixed	MC	0.334
Harris et al. (1977)	69	20.29	NA	NA	NA	p	P&V	paper	16	mixed	MC	0.036
Harris et al. (1977)	110	52.73	NA	NA	NA	p	P&V	paper	16	mixed	MC	1.544
J. T. Kelly and Kelly (1977)	314	39.17	undergrad	NA	USA	p	P&V	paper	12	rect	draw WL	0.548
McGillicuddy-De Lisi et al. (1978)	20	50.00	undergrad	NA	USA	p	P&V	paper	3	jar	draw WL	0.979
Walker and Krasnoff (1978)	88	50.00	undergrad	NA	USA	p	P&V	paper	15	mixed	draw WL	0.439
Liben (1978)	66	50.00	pupils	17.50	USA	p	Scholar	paper	10	mixed	draw WL	0.617
McGiffert Spradlin and Hensley (1979)	120	50.00	undergrad	NA	USA	p	P&V	paper	5	rect	draw WL	1.016
Ray et al. (1979)	135	37.04	undergrad	NA	USA	p	P&V	paper	4	rect	draw WL	0.306
Morgan (1979)	102	50.98	pupils	15.48	USA	np	Pro- Quest	paper	6	florence flask	draw WL	0.806
Treagust (1980)	108	50.00	pupils	15.80	USA	p	P&V	paper	NA	rect	draw WL	0.859
Liben and Golbeck (1980)	240	50.00	pupils	12.62	USA	p	Scholar	paper	6	rect	draw WL	0.469
Thompson et al. (1981)	136	19.19	undergrad	20.00	USA	p	P&V	paper	5	pitcher	draw WL	0.522
Walker et al. (1981)	129	48.84	other	44.12	USA	p	P&V	paper	12	rect	draw WL	0.152
Walker et al. (1981)	129	48.06	pupils	16.66	USA	p	P&V	paper	12	rect	draw WL	0.668
Pearson (1982)	350	43.71	undergrad	NA	USA	np	Scholar	paper	12	mixed	MC	0.775
A. Krasnoff and Walker (1983)	213	53.99	undergrad	NA	USA	p	P&V	paper	12	rect	draw WL	0.411
Linn and Pulos (1983)	778	51.80	pupils	14.89	USA	p	P&V	paper	8	rect	draw WL	0.376
Tegano and Faulkender (1983)	113	37.17	undergrad	NA	USA	p	P&V	paper	2	NA	draw WL	0.808
Kenyon (1984)	30	50.00	undergrad	NA	Canada	p	P&V	paper	8	cylindrical	draw WL	1.383
Liben and Golbeck (1984)	40	50.00	undergrad	19.25	USA	p	P&V	paper	6	rect	draw WL	0.878
Liben and Golbeck (1984)	40	50.00	undergrad	19.25	USA	p	P&V	paper	6	rect	draw WL	0.615
Liben and Golbeck (1984)	80	50.00	undergrad	19.55	USA	p	P&V	paper	6	rect	draw WL	0.854
E. M. Mowrer-Popiel and DeLisi (1984)	125	31.20	undergrad	23.00	USA	p	P&V	paper	11	NA	draw WL	0.493
Myer and Hensley (1984)	85	48.24	undergrad	19.00	USA	p	P&V	paper	2	rect	draw WL	0.554
Wittig and Allen (1984)	195	37.95	undergrad	18.80	USA	p	P&V	mixed	72	mixed	mixed	0.573
Golbeck (1986)	64	50.00	undergrad	21.00	USA	p	P&V	paper	6	rect	draw WL	0.721
Kalichman (1986)	50	50.00	undergrad	NA	USA	p	P&V	paper	8	mixed	draw WL	1.231
Kalichman (1986)	50	50.00	undergrad	NA	USA	p	P&V	paper	8	mixed	draw WL	1.047
Kalichman (1986)	50	50.00	undergrad	NA	USA	p	P&V	paper	8	mixed	draw WL	1.271
Kalichman (1986)	50	50.00	undergrad	NA	USA	p	P&V	paper	8	mixed	draw WL	1.123
Kalichman (1986)	50	50.00	undergrad	NA	USA	p	P&V	paper	8	mixed	draw WL	1.254
Liben and Golbeck (1986)	40	50.00	undergrad	20.00	USA	p	P&V	paper	6	rect	draw WL	0.697
Meehan and Overton (1986)	84	50.00	undergrad	18.92	USA	p	P&V	paper	6	rect	draw WL	0.531
Meehan and Overton (1986)	30	40.00	undergrad	21.25	USA	p	P&V	paper	6	rect	draw WL	0.567
Murphy-Berman et al. (1986)	15	46.67	pupils	10.28	USA	p	P&V	paper	24	rect	draw WL	0.730
Murphy-Berman et al. (1986)	15	46.67	pupils	10.28	USA	p	P&V	paper	24	bottle	draw WL	1.191
Sanders and Ross-Field (1986)	16	50.00	undergrad	25.00	England	p	P&V	paper	10	rect	draw WL	1.701
Batey (1986)	23	65.22	pupils	15.50	USA	np	Pro- Quest	paper	20	mixed	draw WL	−0.357
Batey (1986)	153	52.94	pupils	13.00	USA	np	Pro- Quest	paper	20	mixed	draw WL	1.048
Otani and Leonard (1988)	153	24.18	undergrad	NA	USA	p	P&V	paper	6	mixed	draw WL	0.440
Kalichman (1989a)	194	50.00	undergrad	NA	USA	p	P&V	paper	3	rect	draw WL	0.788
Kalichman (1989b)	104	50.00	undergrad	NA	USA	p	P&V	paper	3	mixed	draw WL	0.522
A. G. Krasnoff et al. (1989)	80	50.00	undergrad	NA	USA	p	P&V	paper	12	rect	draw WL	0.846
Robert and Chaperon (1989)	1520	32.11	undergrad	20.00	Canada	p	P&V	paper	1	cylindrical	draw WL	0.542
Sholl (1989)	477	33.75	undergrad	NA	USA	p	P&V	paper	16	mixed	draw WL	0.446
Signorella et al. (1989)	344	57.56	undergrad	NA	USA	p	P&V	paper	4	rect	draw WL	0.533
Beatty and Duncan (1990)	120	49.17	undergrad	22.95	USA	p	P&V	paper	8	rect	draw WL	0.545
Gladue et al. (1990)	31	51.61	general population	27.81	USA	p	P&V	paper	8	rect	draw WL	0.815
E. Mowrer-Popiel (1990)	140	50.00	other	20.00	USA	np	P&V	paper	6	florence flask	draw WL	0.305
Robert (1990)	284	20.77	undergrad	21.95	Canada	p	P&V	paper	8	mixed	draw WL	0.738
Robert and Tanguay (1990)	32	53.13	other	50.50	Canada	p	P&V	paper	8	mixed	draw WL	0.405
Robert and Tanguay (1990)	28	46.43	other	67.00	Canada	p	P&V	paper	8	mixed	draw WL	0.364
Robert and Tanguay (1990)	30	50.00	other	79.00	Canada	p	P&V	paper	8	mixed	draw WL	1.292
Richa (1990)	60	50.00	other	5.00	India	np	Scholar	real vessel	6	jar	adjust vessel	0.708
Richa (1990)	60	50.00	pupils	10.00	India	np	Scholar	real vessel	6	jar	adjust vessel	0.686
Richa (1990)	60	50.00	pupils	16.50	India	np	Scholar	real vessel	6	jar	adjust vessel	0.707
Lewis and Harris (1990)	56	50.00	undergrad	18.50	USA	p	Scholar	paper	4	mixed	MC	0.428
Kalichman (1991)	76	50.00	undergrad	NA	USA	p	P&V	paper	3	rect	draw WL	1.006
Liben (1991a)	104	48.08	undergrad	19.00	USA	p	P&V	paper	6	rect	draw WL	0.506
Liben (1991a)	200	50.00	undergrad	19.00	USA	p	P&V	paper	6	rect	draw WL	0.453
McAfee and Proffitt (1991)	442	48.42	undergrad	NA	USA	p	P&V	paper	1	rect	draw WL	0.687
McAfee and Proffitt (1991)	53	35.85	undergrad	NA	USA	p	P&V	paper	24	mixed	draw WL	0.310
McCormick and Witelson (1991)	76	50.00	other	25.00	Canada	p	P&V	paper	6	rect	draw WL	1.056
Pennings (1991)	16	50.00	NA	7.90	Netherlands	p	P&V	paper	10	mixed	draw WL	0.741
Pennings (1991)	16	50.00	NA	7.90	Netherlands	p	P&V	paper	10	mixed	draw WL	0.651
Pennings (1991)	16	50.00	NA	7.90	Netherlands	p	P&V	paper	10	mixed	draw WL	0.941
Robert and Morin (1993)	99	37.37	undergrad	21.60	Canada	p	P&V	paper	4	mixed	draw WL	0.516
Thomas and Lohaus (1993)	38	47.37	pupils	6.50	Germany	p	P&V	paper	8	rect	draw WL	0.487
Thomas and Lohaus (1993)	55	54.55	pupils	8.00	Germany	p	P&V	paper	8	rect	draw WL	0.470
Thomas and Lohaus (1993)	35	40.00	pupils	9.00	Germany	p	P&V	paper	8	rect	draw WL	0.197
Thomas and Lohaus (1993)	33	63.64	pupils	10.00	Germany	p	P&V	paper	8	rect	draw WL	0.560
Thomas and Lohaus (1993)	63	38.01	pupils	11.00	Germany	p	P&V	paper	8	rect	draw WL	0.480
Thomas and Lohaus (1993)	81	53.09	pupils	12.00	Germany	p	P&V	paper	8	rect	draw WL	0.872
Thomas and Lohaus (1993)	88	52.23	pupils	13.00	Germany	p	P&V	paper	8	rect	draw WL	0.417
Thomas and Lohaus (1993)	58	53.45	pupils	14.00	Germany	p	P&V	paper	8	rect	draw WL	0.253
Thomas and Lohaus (1993)	67	41.79	pupils	15.00	Germany	p	P&V	paper	8	rect	draw WL	0.275
Thomas and Lohaus (1993)	48	54.17	pupils	16.50	Germany	p	P&V	paper	8	rect	draw WL	−0.054
Thomas and Lohaus (1993)	32	50.00	pupils	9.50	Germany	p	P&V	screen	7	rect	adjust WL	0.776
Thomas and Lohaus (1993)	36	55.56	pupils	11.50	Germany	p	P&V	screen	7	rect	adjust WL	0.462
Thomas and Lohaus (1993)	53	58.49	pupils	13.50	Germany	p	P&V	screen	7	rect	adjust WL	0.489
Thomas and Lohaus (1993)	52	57.69	pupils	16.00	Germany	p	P&V	screen	7	rect	adjust WL	0.531
Vasta et al. (1993)	104	46.15	undergrad	19.40	USA	p	P&V	paper	2	rect	MC	0.652
Lawton (1994)	426	32.39	undergrad	24.30	USA	p	P&V	paper	12	mixed	MC	0.807
Robert and Ohlmann (1994)	280	50.00	undergrad	21.20	Canada	p	P&V	paper	8	rect	draw WL	0.721
Robert et al. (1994)	132	45.45	undergrad	23.30	Canada	p	P&V	paper	NA	rect	draw WL	1.217
Agruso (1994)	268	59.70		17.00	USA	np	Scholar	paper	6	rect	draw WL	0.604
Hammer et al. (1995)	54	50.00	undergrad	20.00	USA	p	P&V	photo	6	bottle	draw WL	1.837
Hammer et al. (1995)	54	50.00	undergrad	25.00	USA	p	P&V	photo	6	bottle	draw WL	0.000
Hecht and Proffitt (1995)	40	40.00	undergrad	25.00	Germany	p	P&V	paper	1	rect	draw WL	0.423
Hecht and Proffitt (1995)	40	40.00	other	38.00	Germany	p	P&V	paper	1	rect	draw WL	0.382
Hecht and Proffitt (1995)	40	40.00	other	51.50	Germany	p	P&V	paper	1	rect	draw WL	1.073
Parameswaran (1995)	122	28.69	undergrad	22.00	USA	p	P&V	paper	7	rect	draw WL	0.413
Sholl and Liben (1995)	177	29.38	undergrad	NA	USA	p	P&V	paper	8	mixed	draw WL	0.693
Sholl and Liben (1995)	669	30.34	undergrad	NA	USA	p	P&V	paper	6	bottle	draw WL	0.536
Sholl and Liben (1995)	902	42.02	undergrad	NA	USA	p	P&V	paper	6	bottle	draw WL	0.729
Wegesin (1995)	80	50.00	other	28.75	USA	np	P&V	paper	20	rect	draw WL	0.731
Lohaus et al. (1996)	202	47.52	pupils	8.14	Germany	p	P&V	paper	8	rect	draw WL	0.202
Lohaus et al. (1996)	194	53.61	pupils	11.28	Germany	p	P&V	paper	8	rect	draw WL	0.470
Lohaus et al. (1996)	202	54.46	pupils	14.20	Germany	p	P&V	paper	8	rect	draw WL	0.794
Parameswaran and De Lisi (1996)	122	28.69	undergrad	21.90	USA	p	P&V	paper	7	rect	draw WL	0.413
Robert and Harel (1996)	520	55.77	undergrad	22.50	Canada	p	P&V	paper	8	mixed	draw WL	0.671
Robert and Harel (1996)	466	42.92	undergrad	22.50	Canada	p	P&V	paper	8	mixed	draw WL	0.502
Robert and Harel (1996)	426	45.54	undergrad	22.50	Canada	p	P&V	paper	8	mixed	draw WL	0.657
Vasta et al. (1996)	180	46.67	undergrad	21.05	USA	p	P&V	paper	16	rect	draw WL	0.016
Voyer (1996)	125	56.80	undergrad	19.40	Canada	p	P&V	paper	16	rect	draw WL	0.644
Farace (1996)	155	51.61	other	20.24	USA	np	Scholar	paper	10	jar	draw WL	0.611
Merriwether and Liben (1997)	160	50.00	undergrad	18.90	USA	p	P&V	paper	6	rect	draw WL	0.463
Pulos (1997)	80	50.00	undergrad	NA	USA	p	P&V	paper	4	rect	draw WL	0.324
Pulos (1997)	80	50.00	undergrad	NA	USA	p	P&V	paper	4	rect	draw WL	0.256
Dessens et al. (1998)	142	49.30	healthy control group	32.50	Nether-lands	p	P&V	paper	NA	rect	draw WL	0.627
Wegesin (1998)	40	50.00	other	25.05	USA	p	P&V	paper	20	rect	draw WL	0.727
Voyer (1998)	98	46.94	undergrad	21.20	Canada	p	Scholar	paper	16	mixed	draw WL	0.773
Silver (1998)	52	50.00	other	26.00	USA	p	Scholar	paper	1	bottle	draw WL	0.380
Silver (1998)	66	50.00	pupils	13.50	USA	p	Scholar	paper	1	bottle	draw WL	0.140
Silver (1998)	52	51.92	pupils	10.00	USA	p	Scholar	paper	1	bottle	draw WL	0.074
Nordvik and Amponsah (1998)	161	58.39	undergrad	20.05	Norway	p	Scholar	paper	6	mixed	MC	0.809
Nordvik and Amponsah (1998)	293	23.21	undergrad	22.10	Norway	p	Scholar	paper	6	mixed	MC	0.395
Epting and Overman (1998)	47	42.55	undergrad	20.08	USA	p	Scholar	paper	8	rect	draw WL	1.093
Li et al. (1999a)	485	52.67	pupils	13.14	China	p	P&V	paper	8	bottle	draw WL	0.281
Li et al. (1999b)	295	36.27	undergrad	20.00	China	p	P&V	paper	8	bottle	draw WL	0.648
Li et al. (1999b)	49	42.86	undergrad	19.00	USA	p	P&V	paper	8	bottle	draw WL	0.483
Li et al. (1999b)	129	46.51	undergrad	19.00	USA	p	P&V	paper	8	bottle	draw WL	0.053
Neave et al. (1999)	62	54.84	other	28.67	England	p	Scholar	paper	15	rect	draw WL	0.288
Lerner (1999)	160	44.00	pupils	11.80	USA	p	Scholar	paper	6	NA	draw WL	0.439
Lerner (1999)	242	49.00	pupils	12.30	Serbia	p	Scholar	paper	6	NA	draw WL	0.291
Herlitz et al. (1999)	200	50.00	general population	28.10	Sweden	p	Scholar	paper	8	NA	draw WL	0.039
Amponsah (2000)	266	56.02	pupils	12.00	Ghana	p	P&V	paper	6	rect	MC	0.463
Amponsah (2000)	303	49.91	pupils	16.00	Ghana	p	P&V	paper	6	rect	MC	0.383
Amponsah (2000)	390	52.82	undergrad	NA	Ghana	p	P&V	paper	6	rect	MC	0.631
Amponsah (2000)	297	48.15	pupils	12.00	Norway	p	P&V	paper	6	rect	MC	0.690
Amponsah (2000)	310	44.84	pupils	16.00	Norway	p	P&V	paper	6	rect	MC	0.790
Amponsah (2000)	430	35.81	undergrad	NA	Norway	p	P&V	paper	6	rect	MC	0.594
Amponsah (2000)	249	50.60	pupils	9.00	Ghana	p	P&V	paper	16	rect	MC	0.335
Amponsah (2000)	193	48.70	pupils	9.00	Norway	p	P&V	paper	16	rect	MC	0.327
Amponsah and Krekling (1994)	417	35.73	undergrad	26.00	Ghana and Norway	p	WoS	paper	9	mixed	MC	0.557
Golbeck and Sinagra (2000)	25	0.24	undergrad	22.70	USA	p	P&V	paper	6	rect	draw WL	0.109
Voyer et al. (2000)	344	42.44	undergrad	19.40	Canada	p	P&V	paper	16	mixed	draw WL	0.571
Lucas and Alwens (2000)	26	46.15	undergrad	NA	USA	np	Scholar	screen	8	NA	draw WL	1.153
Lucas and Alwens (2000)	17	47.06	undergrad	NA	USA	np	Scholar	screen	8	NA	draw WL	−0.354
Lucas and Alwens (2000)	24	50.00	undergrad	NA	USA	np	Scholar	screen	8	NA	draw WL	0.630
Lucas and Alwens (2000)	16	50.00	undergrad	NA	USA	np	Scholar	screen	8	NA	draw WL	0.276
Beisteiner (2001)	1022	54.99	NA	22.97	Austria	np	P&V	paper	12	rect	draw WL	0.277
Casey et al. (2001)	187	48.66	pupils	13.83	USA	p	P&V	paper	8	rect	draw WL	0.670
Quaiser-Pohl (2001)	444	49.77	pupils	9.62	Germany	p	P&V	paper	12	rect	draw WL	0.394
Roberts and Bell (2002)	64	50.00	other	14.04	USA	p	P&V	paper	7	rect	draw WL	0.606
Lewin et al. (2001)	185	49.19	general population	29.37	Sweden	p	Scopus	paper	10	cylindrical	MC	0.436
Levine and Stern (2002)	89	35.96	undergrad	20.00	USA	p	P&V	paper	6	rect	draw WL	0.616
Seng and Tan (2002)	57	55.36	pupils	10.00	China	np	P&V	paper	8	bottle	draw WL	0.192
Seng and Tan (2002)	38	60.26	pupils	10.00	Malaysia	np	P&V	paper	8	bottle	draw WL	0.347
Seng and Tan (2002)	5	0.40	pupils	10.00	Singapore	np	P&V	paper	8	bottle	draw WL	1.006
Robert and Berthiaume (2002)	46	50.00	other	17.65	Canada	p	Scholar	screen	30	rect	draw WL	0.730
Robert and Berthiaume (2002)	46	50.00	other	17.65	Canada	p	Scholar	screen	240	rect	correct WL yes/no	0.286
Robert and Berthiaume (2002)	46	50.00	other	17.65	Canada	p	Scholar	paper	NA	rect	draw WL	0.057
Robert and Berthiaume (2002)	46	50.00	other	17.65	Canada	p	Scholar	paper	NA	rect	correct WL yes/no	0.460
Formann (2003)	431	52.67	general population	NA	Austria	p	P&V	paper	12	florence flask	draw WL	0.190
Loehlin and McFadden (2003)	255	41.57	other	21.91	USA	p	P&V	paper	NA	rect	draw WL	0.329
Voyer and Sullivan (2003)	140	39.29	undergrad	19.50	Canada	p	P&V	paper	16	rect	draw WL	0.782
Voyer and Sullivan (2003)	119	42.86	undergrad	19.50	Canada	p	P&V	paper	16	rect	draw WL	0.453
Voyer and Sullivan (2003)	195	46.15	undergrad	19.50	Canada	p	P&V	paper	16	rect	draw WL	0.582
Voyer and Sullivan (2003)	48	43.75	undergrad	19.50	Canada	p	P&V	paper	16	rect	draw WL	1.155
Hill (2004)	69	47.83	undergrad	20.23	USA	np	Cited- Ref	paper	4	rect	draw WL	0.987
Fernández (2004) ^a^	80	61.14	pupils	10.23	Spain	p	Psyc- Info	paper	6	mixed	draw WL	0.087
Robert and Héroux (2004)	37	54.05	pupils	9.92	Canada	p	P&V	paper	6	rect	draw WL	0.128
Robert and Héroux (2004)	41	46.34	pupils	12.83	Canada	p	P&V	paper	6	rect	draw WL	0.210
Robert and Héroux (2004)	36	50.00	pupils	15.83	Canada	p	P&V	paper	6	rect	draw WL	0.221
Ecuyer-Dab and Robert (2004)	216	43.98	general population	34.25	Canada	p	Scholar	paper	8	rect	draw WL	0.769
Ansel (2004)	143	48.25	pupils	12.50	USA	np	Scholar	paper	2	rect	draw WL	0.256
Robert and Longpré (2005)	80	50.00	undergrad	23.60	Canada	p	P&V	paper	10	rect	draw WL	0.949
Verdine (2005)	16	50.00	healthy control group	6.20	USA	np	Scholar	paper	4	rect	MC	1.192
Frick (2005)	80	50.00	other	12.06	Switzer-land	np	Scholar	paper	8	rect	draw WL	0.475
Alexander (2005)	120	50.00	undergrad	20.40	USA	p	Scholar	paper	10	jar	draw WL	0.700
Neumann et al. (2007)	64	50.00	undergrad	19.98	Australia	p	P&V	screen	15	glass/cup	draw WL	0.606
Cherney et al. (2008)	124	48.39	undergrad	19.30	USA	p	P&V	paper	8	rect	draw WL	0.333
Morra (2008)	337	51.04	pupils	8.92	Italy	p	P&V	paper	8	rect	draw WL	0.231
Rilea (2008)	94	50.00	undergrad	NA	USA	p	P&V	screen	216	rect	MC	0.904
Tran and Formann (2008)	147	51.70	general population	NA	Austria	p	P&V	paper	8	florence flask	draw WL	0.243
Tran and Formann (2008)	296	52.70	general population	NA	Austria	p	P&V	paper	8	florence flask	draw WL	0.006
Tran and Formann (2008)	1214	44.65	general population	NA	Austria	p	P&V	paper	8	florence flask	draw WL	0.592
Jansen-Osmann et al. (2008) ^a^	20	35.00	pupils	11.80	Germany	p	WoS	paper	NA	rect	draw WL	0.923
Weixlbraun (2009)	143	48.95	general population	34.38	Austria	np	OATD	paper	8	florence flask	draw WL	0.860
Liben et al. (2010)	49	42.86	undergrad	18.50	USA	p	Cited- Ref	paper	6	rect	Draw WL	0.328
Liben et al. (2011)	582	37.29	undergrad	NA	USA	p	Scholar	paper	6	rect	draw WL	0.261
Christensen (2011)	58	51.72	other	10.03	USA	np	Pro- Quest	paper	6	rect	draw WL	0.315
Berenbaum et al. (2012)	27	51.85	healthy control group	19.47	USA	p	Scholar	paper	12	bottle	MC	0.873
Cashdan et al. (2012)	103	50.49	other	NA	Tanzania	p	WoS	paper	1	glass/cup	MC	0.606
Doyle et al. (2012)	403	33.00	undergrad	19.63	USA and Canada	p	WoS	paper	16	mixed	draw WL	0.540
Al-Balushi and Al-Battashi (2013)	101	53.47	pupils	14.50	Oman	p	Scholar	paper	8	bottle	draw WL	0.413
Lange-Küttner and Ebersbach (2013)	18	44.44	pupils	6.58	Germany	p	Scholar	paper	7	NA	draw WL	0.381
Lange-Küttner and Ebersbach (2013)	23	47.83	pupils	7.33	Germany	p	Scholar	paper	7	NA	draw WL	0.045
Lange-Küttner and Ebersbach (2013)	37	54.04	pupils	8.50	Germany	p	Scholar	paper	7	NA	draw WL	−0.256
Lange-Küttner and Ebersbach (2013)	19	52.63	pupils	9.50	Germany	p	Scholar	paper	7	NA	draw WL	−0.521
Ruwisch and Lüthje (2013) ^a^	65	61.54	other	6.25	Germany	p	Cited- Ref	photo	1	bottle	MC	0.212
Gagatsis and Kalogirou (2013) ^a^	350	52.29	pupils	12.33	Cyprus	np	Cited- Ref	paper	1	rect	MC	0.220
S. M. Weisberg et al. (2014)	72	50.00	undergrad	22.40	USA	p	WoS	paper	NA	rect	draw WL	0.420
S. Weisberg and Newcombe (2014)	52	50.00	undergrad	NA	USA	p	Scholar	paper	6	bottle	Draw WL	0.020
Nardi et al. (2014)	40	50.00	undergrad	25.50	Italy	p	Cited- Ref	paper	6	rect	draw WL	0.184
Holden et al. (2015)	81	39.51	undergrad	21.49	England	p	WoS	paper	13	rect	draw WL	0.457
Vashro and Cashdan (2015)	122	54.92	other	NA	Namibia	p	Scholar	paper	1	glass/cup	MC	0.429
Coyle (2015)	61	49.18	other	5.17	USA	np	Pro- Quest	paper	6	jar	MC	−0.384
Merrill et al. (2016)	153	52.29	pupils	9.62	USA	p	WoS	paper	12	jar	draw WL	0.367
Reilly et al. (2016)	309	33.98	undergrad	25.46	Australia	p	Scholar	screen	10	NA	draw WL	0.258
Goldsmith et al. (2016) ^a^	53	69.81	pupils	14.50	USA	p	Scopus	paper	6	rect	draw WL	−0.559
Goldsmith et al. (2016) ^a^	50	78.00	pupils	14.50	USA	p	Scopus	paper	6	rect	draw WL	0.655
Goldsmith et al. (2016) ^a^	28	64.29	pupils	14.50	USA	p	Scopus	paper	6	rect	draw WL	−0.400
Hannula (2019)	82	64.63	undergrad	21.00	Canada	p	Scholar	paper	6	bottle	draw WL	0.290
S. A. Sorby and Veurink (2019)	71	47.89	undergrad	12.50	USA	p	Scopus	paper	6	rect	draw WL	0.320
S. A. Sorby and Veurink (2019)	34	47.06	undergrad	12.50	USA	p	Scopus	paper	6	rect	draw WL	0.421
S. A. Sorby and Veurink (2019)	72	47.22	pupils	12.50	USA	p	Scopus	paper	6	rect	draw WL	−0.493
Smith (2019)	304	35.86	pupils	21.40	Canada	np	Cited- Ref	screen	6	glass/cup	MC	0.583
Barhorst-Cates et al. (2020)	72	49.30	pupils	9.28	USA	p	WoS	paper	8	rect	draw WL	0.324
Côté and Lalumière (2020)	318	37.11	undergrad	42.60	Canada	p	Scholar	paper	8	rect	draw WL	0.941
Côté and Lalumière (2020)	131	49.62	pupils	40.90	Canada	p	Scholar	paper	8	rect	draw WL	0.567
Brase (2021)	304	29.27	other	19.96	USA	p	Scholar	screen	7	jar	MC	0.319
Meneghetti et al. (2021)	52	65.38	other	19.10	Italy	p	Cited- Ref	paper	16	mixed	draw WL	0.461
Meneghetti et al. (2021)	45	60.00	undergrad	20.20	Italy	p	Cited- Ref	paper	16	mixed	draw WL	0.576
Taufer (2021)	32	56.25	other	24.53	Italy	np	Cited- Ref	screen	8	bottle	MC	0.379
Davis et al. (2021)	83	34.48	pupils	11.68	Namibia	p	WoS	screen	1	glass/cup	MC	−0.261
Jansen and Pietsch (2022)	92	54.34	other	9.03	Germany	p	Scholar	paper	16	mixed	draw WL	0.455
D. Kelly and Beltz (2022)	336	33.63	other	19.23	USA	p	Scholar	screen	12	jar	MC	0.432
Rahe and Jansen (2022)	117	38.46	pupils	26.30	Germany	p	WoS	screen	3	bucket	MC	0.467
Kim and Li (2022) ^a^	20	50.00	undergrad	21.35	USA	p	Cited- Ref	paper	10	rect	draw WL	0.454
Polifka et al. (2023)	174	35.63	NA	19.93	USA	p	Scholar	screen	3	rect	draw WL	0.490
Xia and Chen (2023)	30	43.33	undergrad	6.00	China	np	Scholar	screen	4	rect	MC	1.420
Tomai et al. (2024)	28	67.86	undergrad	49.32	Greece	p	Scopus	paper	7	rect	draw WL	0.289
Tomai et al. (2024)	30	30.00	pupils	47.40	Greece	p	Scopus	paper	7	rect	draw WL	0.260
Scott et al. (2023) ^a^	154	44.81	other	8.04	England	p	Scholar	paper	16	mixed	draw WL	0.391
Bracken et al. (2024)	36	38.89	other	5.43	USA	p	WoS	screen	11	jar	draw WL	−0.112
Morawietz et al. (2024c)	25	52.00	pupils	10.70	Germany	p	Scopus	paper	12	jar	draw WL	0.174
Morawietz et al. (2024c)	24	50.00	healthy control group	11.40	Germany	p	Scopus	paper	12	jar	draw WL	1.011
Signorella and Liben (2024)	290	51.03	pupils	19.00	USA	p	Scopus	screen	15	mixed	correct WL yes/no	0.372
McNeal et al. (2024) ^a^	71	64.79	pupils	NA	USA	p	Scopus	paper	6	bottle	draw WL	0.237
Morawietz et al. (2024a) ^a^	26	50.00	other	11.40	Germany	p	WoS	paper	12	jar	draw WL	−0.310
Morawietz et al. (2024a) ^a^	22	40.91	undergrad	12.05	Germany	p	WoS	paper	12	jar	draw WL	0.881
Morawietz et al. (2024a) ^a^	26	57.69	pupils	26.10	Germany	p	WoS	paper	12	jar	draw WL	0.159
Morawietz et al. (2024b) ^a^	27	44.44	pupils	11.30	Germany	p	Scholar	paper	12	jar	draw WL	−0.110
Morawietz et al. (2024b) ^a^	26	42.31	undergrad	11.30	Germany	p	Scholar	paper	12	jar	draw WL	−0.312
Lasc et al. (2025)	171	43.86	pupils	6.81	USA	p	Cited- Ref	paper	11	jar	draw WL	0.278
Feraco (2025) ^a^	45	57.78	pupils	28.60	Italy	np	Author	screen	8	bottle	MC	1.314
Feraco (2025) ^a^	45	35.56	other	21.00	Italy	np	Author	screen	8	bottle	MC	1.290
Signorella (2025) ^a^	121	42.98	undergrad	NA	USA	np	Author	paper	1	bottle	draw WL	0.521

Note. Undergrad = undergraduate students; Origin = participant country of origin; Pub = publication status (p = published; np = not published); Source = data source (P&V = Pietschnig & Voracek, 2011; Scholar = Google Scholar; WoS = Web of Science; CitedRef = forward citation search of previous meta-analyses; Author = unpublished data sent by the author); paper = stimuli presented on paper; screen = stimuli presented on a screen; photo = stimuli were photos; real vessel = stimuli were models; Shape = shape of the vessel (rect = rectangular; mixed = mixed shapes); MC = multiple choice; WL = Water level; NA = information not available. ^a^ Information used to compute the effect size was received through personal communication.

**Table 2:** Results of overall and subgroup analyses.

	Summary Effect (*d*)	SE	95% CI	95% PI	*p*-Value	*U*_1_	*TR*_2SD_	*Q*	*I*^2^ (%)	τ
Overall (*k* = 267)	0.501	0.018	[0.465, 0.537]	[0.120, 0.882]	<.001	0.33	2.94	593.97 ^***^	52.9	0.19
Response format										
Water level drawing (*k* = 211)	0.481	0.021	[0.440, 0.522]	[0.106, 0.857]	<.001	0.32	2.83	460.60 ^***^	51.3	0.19
Multiple choice (*k* = 39)	0.580	0.052	[0.478, 0.682]	[0.068, 1.092]	<.001	0.37	3.42	115.82 ^***^	72.2	0.26
Water level adjusting (*k* = 10)	0.526	0.086	[0.357, 0.694]	[0.357, 0.694]	<.001	0.34	3.09	0.83	0.0	0.00
Test setting										
Group (*k* = 118)	0.520	0.027	[0.468, 0.572]	[0.132, 0.907]	<.001	0.34	3.05	312.96 ^***^	59.2	0.20
Individual (*k* = 99)	0.524	0.032	[0.462, 0.586]	[0.180, 0.869]	<.001	0.34	3.08	157.06 ^***^	35.1	0.17
Publication status										
Published (*k* = 224)	0.495	0.019	[0.458, 0.533]	[0.133, 0.858]	<.001	0.33	2.91	489.23 ^***^	51.5	0.18
Unpublished (*k* = 43)	0.540	0.059	[0.425, 0.655]	[0.009, 1.071]	<.001	0.35	3.17	104.64 ^***^	61.1	0.26
Horizontal reference line										
Provided (*k* = 148)	0.463	0.026	[0.412, 0.514]	[0.047, 0.878]	<.001	0.31	2.73	326.70 ^***^	56.9	0.21
Not provided (*k* = 45)	0.526	0.033	[0.461, 0.591]	[0.313, 0.738]	<.001	0.34	3.09	65.13 ^*^	24.2	0.10
Sex of first author										
Men (*k* = 117)	0.505	0.027	[0.452, 0.557]	[0.127, 0.882]	<.001	0.33	2.96	283.70 ^***^	53.8	0.19
Women (*k* = 143)	0.498	0.026	[0.468, 0.550]	[0.093, 0.904]	<.001	0.33	2.93	301.32 ^***^	54.8	0.21
Vessel shape										
Rectangular (*k* = 129)	0.504	0.025	[0.455, 0.554]	[0.135, 0.874]	<.001	0.33	2.96	294.30 ^***^	52.3	0.19
Cylindrical (*k* = 6)	0.572	0.109	[0.358, 0.785]	[0.159, 0.984]	<.001	0.37	3.37	11.92 ^*^	54.3	0.18
Florence flask (*k* = 12)	0.435	0.085	[0.268, 0.602]	[−0.033, 0.903]	<.001	0.29	2.58	37.17 ^***^	68.4	0.22
Bottle (*k* = 31)	0.438	0.062	[0.318, 0.559]	[0.053, 0.823]	<.001	0.30	2.60	55.70 ^**^	40.9	0.19
Jar (*k* = 19)	0.375	0.079	[0.219, 0.531]	[−0.085, 0.834]	<.001	0.26	2.29	33.99 ^*^	47.8	0.22
Cup (*k* = 5)	0.500	0.083	[0.338, 0.663]	[0.338, 0.663]	<.001	0.33	2.94	6.77	0.0	0.00
Mixed shapes (*k* = 47)	0.624	0.038	[0.549, 0.699]	[0.307, 0.942]	<.001	0.39	3.71	82.70 ^***^	43.9	0.16
Presentation mode										
Paper (*k* = 229)	0.498	0.020	[0.459, 0.538]	[0.103, 0.894]	<.001	0.33	2.93	539.26 ^***^	56.1	0.20
Screen (*k* = 24)	0.517	0.058	[0.403, 0.631]	[0.204, 0.829]	<.001	0.34	3.03	38.54 ^*^	32.2	0.15
Photograph (*k* = 3)	0.591	0.539	[−0.464, 1.648]	[−1.323, 2.506]	.273	0.38	3.49	7.53 ^*^	77.4	0.81
Real vessel (*k* = 9)	0.573	0.086	[0.406, 0.741]	[0.405, 0.741]	<.001	0.37	3.38	1.30	0.0	0.00
Power vs speed test										
Power (*k* = 221)	0.506	0.020	[0.467, 0.545]	[0.133, 0.879]	<.001	0.33	2.97	486.06 ^***^	52.0	0.19
Speed (*k* = 39)	0.507	0.057	[0.400, 0.614]	[0.029, 0.986]	<.001	0.33	2.98	89.26 ^***^	62.3	0.24
Sample origin										
North America (*k* = 170)	0.524	0.023	[0.480, 0.568]	[0.160, 0.888]	<.001	0.34	3.08	376.54 ^***^	49.0	0.18
Europe (*k* = 71)	0.427	0.035	[0.357, 0.496]	[0.066, 0.788]	<.001	0.29	2.54	140.08 ^***^	49.3	0.18
Asia (*k* = 10)	0.507	0.097	[0.317, 0.697]	[0.136, 0.878]	<.001	0.33	2.98	13.24	31.6	0.16
Africa (*k* = 8)	0.472	0.052	[0.370, 0.573]	[0.326, 0.617]	<.001	0.31	2.78	10.52	13.3	0.05
Australia (*k* = 2)	0.359	0.158	[0.023, 0.668]	[−0.058, 0.776]	.023	0.25	2.21	1.51	33.9	0.14
Sample type										
Undergraduates (*k* = 110)	0.567	0.021	[0.525, 0.608]	[0.348, 0.785]	<.001	0.36	3.33	163.31 ^***^	28.7	0.11
Pupils (*k* = 99)	0.401	0.033	[0.338, 0.465]	[0.001, 0.801]	<.001	0.27	2.41	215.14 ^***^	51.6	0.20
General population (*k* = 16)	0.412	0.089	[0.238, 0.586]	[−0.179, 1.002]	<.001	0.28	2.47	60.81 ^***^	77.6	0.29
Healthy control group (*k* = 4)	0.574	0.240	[0.103, 1.045]	[−0.239, 1.387]	.017	0.37	3.38	5.96	51.3	0.34
Other (*k* = 31)	0.521	0.055	[0.413, 0.628]	[0.182, 0.859]	<.001	0.34	3.05	43.68	31.9	0.16
Age group										
Children (*k* = 52)	0.361	0.045	[0.272, 0.450]	[0.008, 0.714]	<.001	0.25	2.22	93.67 ^***^	38.5	0.17
Adolescents (*k* = 56)	0.406	0.048	[0.311, 0.500]	[−0.117, 0.928]	<.001	0.28	2.44	142.00 ^***^	64.4	0.26
Adults (*k* = 152)	0.573	0.020	[0.533, 0.613]	[0.295, 0.851]	<.001	0.37	3.38	250.18 ^***^	38.4	0.14

Note. *SE* = standard error; 95% CI = upper and lower bounds of 95% confidence interval; 95% PI = upper and lower bounds of 95% prediction interval; *Q* = Cochran’s *Q* test statistic for heterogeneity; U_1_ = proportion of nonoverlap relative to the joint distribution of men and women; TR_2SD_ = upper-tail ratio for the distributional region beyond two standard deviation units of the female mean; *I*^2^ = proportion of observed variation reflecting true heterogeneity; τ = estimated standard deviation of true effect sizes. * *p* < .05. ** *p* < .01. *** *p* < .001.

## Data Availability

All data are available from https://osf.io/6fwsa/files/osfstorage, accessed on 28 August 2026.

## Supplementary Materials

The following supporting information can be downloaded at: https://www.mdpi.com/article/10.3390/jintelligence14100243/s1. Table S1. PRISMA 2020 checklist. Table S1.1: Deviations from the pre-registration; Table S2.1: PRISMA 2020 checklist; Table S4.1: Numerical outcomes of dissemination bias analyses; Figure S4.1: *P*-curve plot; Figure S4.2: Contour- and power-enhanced funnel plots; Figure S5.1: Number of included effect sizes per publication year; Figure S5.2: Heat map of sample countries of origin; Figure S5.3: Forest plot of random-effects meta-analysis; Figure S5.4: Results of influence diagnostics; Figure S5.5: Cumulative forest plot according to publication year; Figure S5.6: Histogram of the *p*-value distribution for all meta-analytic specifications; Table S6.1: Sensitivity analysis for unequal variances; Table S7.1: Results of linear precision-weighted meta-regression analyses. All data, the analysis code, and supplementary tables are available at https://osf.io/6fwsa/files/osfstorage (accessed on 28 August 2026).
